# The Burden of Long-COVID-19 Among Pediatric Subjects: A Systematic Review and Meta-Analysis

**DOI:** 10.3390/jcm15145597

**Published:** 2026-07-16

**Authors:** Giovanni Cioni, Giovanna Letizia Calo’, Borana Kerpaci, Anastasia Troia, Natalia Gregori, Lamberto Manzoli, Maria Elena Flacco

**Affiliations:** 1 Department of Environmental and Prevention Sciences, University of Ferrara, Via Fossato di Mortara 64, 44121 Ferrara, Italy; giovanni.cioni@unife.it (G.C.); giovannaletizia.calo@unife.it (G.L.C.); borana.kerpaci@edu.unife.it (B.K.); anastasia.troia@unife.it (A.T.); 2 Department of Medical and Surgical Sciences, University of Bologna, Via San Giacomo 12, 40138 Bologna, Italy; natalia.gregori@studio.unibo.it (N.G.); lmanzoli@post.harvard.edu (L.M.)

**Keywords:** COVID-19, Long-COVID, post COVID-19 condition (PCC), post-acute sequelae of COVID-19 (PASC), Long-COVID symptoms, children, adolescents, meta-analysis

## Abstract

**Background/Objectives**: There is wide variability in the available estimates on the burden of Long-COVID (LC), both among adults and children. Preliminary evidence suggests that this inconsistency may be due, at least in part, to the adoption of uneven diagnostic criteria, proposed by several international entities during the course of the pandemic, but no comprehensive evaluation has been performed so far to quantify the extent of variation among children. This systematic review and meta-analysis aims to update the available epidemiological estimates of LC among pediatric subjects, and to systematically appraise the degree of variability that can be attributed to the diagnostic criteria adopted. **Methods**: A systematic literature search identified cohort studies providing data on LC among children and adolescents with a previous SARS-CoV-2 infection. We computed (a) proportion and (b) head-to-head meta-analyses (a) to quantify the pooled rates of LC and LC-related symptoms, and (b) to assess the likelihood of developing LC based upon selected demographic and/or clinical characteristics, respectively. **Results**: In total, 52 cohort studies and 960,089 subjects were included. The pooled rate of LC was 18.1% (95% CI: 13.1–23.6), ranging from 16.2% to 21.6%, according to NIH or WHO diagnostic criteria, respectively. Fatigue, respiratory and neurological symptoms were most commonly reported by LC patients. Stratified analyses showed that females, adolescents (vs. children), subjects with comorbidities, and those with a previous severe COVID-19 (as compared to asymptomatic primary infections) were more likely to develop LC (all *p* < 0.05). In contrast, the likelihood of LC onset did not significantly vary between individuals with ≥1 anti-SARS-CoV-2 vaccine dose, versus the unvaccinated (pooled OR: 0.92; 95% CI: 0.61–1.41). **Conclusions**: Although the available estimates may largely vary depending on the adopted definition, LC affects a meaningful proportion of the pediatric population; as such, common clinical criteria and diagnostic approaches are urgently needed. Moreover, given the relevant methodological heterogeneity and the generally poor-to-fair quality of a large part of the available literature, high-quality studies adopting standardized methodologies are warranted to identify at-risk populations and guide the implementation of targeted follow-up strategies.

## 1. Introduction

The term “Long-COVID” (LC) defines a complex, multisystem syndrome characterized by the persistence or the new onset of signs and symptoms (such as fatigue, cough, headache, altered smell or anosmia, arthralgia or myalgia, and chest tightness or pain [1]) after a primary SARS-CoV-2 infection. [2]. Initially described only among adults during the early phases of the COVID-19 pandemic [3], this condition was also much later recognized among children and adolescents [4]. Over the course of the pandemic, this disorder has been defined by multiple international expert panels and health organizations, including the World Health Organization (WHO), the National Institute for Health and Care Excellence (NICE), and the National Institutes of Health (NIH), and has been appointed using different names, including Long-COVID (LC), post-acute sequelae of SARS-CoV-2 infection (PASC), and, according to the WHO, post-COVID-19 condition (PCC) [5]. Besides the multiplicity of names and definitions used to describe this condition, considerable heterogeneity exists in the diagnostic criteria proposed to identify this syndrome [6,7,8,9,10,11,12].

Clearly, the absence of a universally accepted definition of LC has largely contributed to the wide discrepancies emerging from the available evidence: current epidemiological estimates widely vary among adults [13], and even more so among children, with prevalence data ranging from 3% to 23% [14,15], according to the diagnostic criteria adopted. It has also been suggested that the type and the severity of the symptoms may vary between infants, school-aged children, and adolescents [16], and depending on the severity of the acute SARS-CoV-2 infection, and the circulating viral variants [17]. Additionally, the pooled estimates so far available have included studies up to the end of 2022 [18], were based upon the combination of prospective and retrospective cohorts, cross-sectional and case–control evaluations, are highly fragmented and heterogeneous, and the results are complex to interpret by examining single studies [19,20,21].

Therefore, we performed a meta-analysis of cohort studies in order to: (a) update the available evidence on the burden of LC among pediatric and adolescent individuals; (b) systematically appraise the degree of variability that can be attributed to the diagnostic criteria adopted; (c) quantify the pooled rates of the main symptoms related to LC; and (d) clarify whether its frequency and severity may vary in the presence of selected demographic and clinical characteristics.

## 2. Materials and Methods

### 2.1. Search Strategy, Selection Criteria and Methodological Approach

The reporting of this meta-analysis followed the Preferred Reporting Items for Systematic Reviews and Meta-Analyses (PRISMA 2020) Statement [22]. The bibliographic search was first performed on Medline and Scopus databases by two investigators independently (G.C., G.L.C.), and the last search update was performed on 14 October 2025. The following search strings were built, adapted to each database: ((Long-COVID[Title/Abstract]) OR (LC[Title/Abstract]) OR (post-COVID-19 condition[Title/Abstract]) OR (PCC[Title/Abstract]) OR (post-acute sequelae of COVID-19[Title/Abstract]) OR (PASC[Title/Abstract])) AND ((child*[Title/Abstract]) OR (adolesc*[Title/Abstract])) for Medline; (TITLE-ABS-KEY(Long-COVID) OR TITLE-ABS-KEY (LC) OR TITLE-ABS-KEY (post-COVID-19 condition) OR TITLE-ABS-KEY (PCC) OR TITLE-ABS-KEY (post-acute sequelae of COVID-19) OR TITLE-ABS-KEY (PASC) AND TITLE-ABS-KEY (child*) OR TITLE-ABS-KEY (adolesc*)) for Scopus. The reference lists of the retrieved articles were also screened for additional pertinent papers. During the process of the literature search, efforts were also made to identify potentially relevant publications, not published in Medline- or Scopus-indexed journals, but available in the grey literature. Accordingly, an extra search was carried out on Google using “Long-COVID”, “children”, and “adolescents” as keywords. Studies were eligible if they met the following criteria: (a) were cohort studies, either prospective or retrospective, including data on pediatric or adolescent subjects (0–18 years) with a previous history of laboratory confirmed SARS-CoV-2 infection, through a positive reverse-transcriptase polymerase chain reaction (RT-PCR) test, and/or a baseline positive serology investigated with the use of an anti-trimeric spike IgG enzyme-linked immunosorbent assay (ELISA) [23]; (b) provided the raw number of LC cases upon the total number of SARS-CoV-2-positive subjects, or provided sufficient data to compute these estimates; (c) clearly reported the definition of LC used, or described a symptom duration compatible with at least one established and validated LC definition, according to: National Institute for Health and Care Excellence (NICE), National Institute of Health (NIH), Children and Young People with Long-COVID (CLoCK) Consortium, Post-COVID Core Outcome Set (PC-COS) Children Study Group, World Health Organization (WHO) or National Academies of Sciences, Engineering, and Medicine (NASEM); (d) reported at least one outcome of interest at a minimum follow-up of ≥4 weeks after an acute infection; and (e) were published in English. The definitions of pediatric Long-COVID proposed over time are reported in Table 1. No eligibility restrictions were applied with respect to clinical setting (hospitalized or community-based populations), geographical region, SARS-CoV-2 variant, baseline disease severity, population characteristics (including comorbidities), or vaccination status. Studies focusing on mixed adult-pediatric subjects were included only if specific data on the pediatric population were provided.

Some of the included studies were based on large national or international cohorts that generated multiple publications from partially or totally overlapping populations (e.g., CLoCK and RECOVER). To minimize the risk of including each population more than once, overlapping publications were identified by cross-checking cohort name, country, recruitment period and study design. When multiple reports referred to the same cohort, a single study was selected for each analysis according to predefined criteria. For the overall estimates, priority was given to studies with the longest follow-up to better capture long-term outcomes. In cases of identical follow-up length, the study with the largest sample size and most complete outcome reporting was selected. For subgroup and head-to-head meta-analyses, study selection additionally considered the relevance of the reported data to the specific comparison or outcome of interest. Discrepancies in study selection or in data extraction were resolved through discussion with a senior author (M.E.F.). This process was applied to each analysis rate to ensure consistency. The current scenario of the available overlapping cohorts, the associated publications, and the study included in each specific analysis has been summarized in Table S2.

### 2.2. Data Analysis

We first performed random-effects meta-analyses of proportions to obtain pooled rates of LC in the overall sample, and stratified by: (a) adopted LC definitions (the definitions from the CLOCK Consortium and PC-COS were analyzed together, like those from the NIH and NASEM, as they represent two sets of definitions that are similar in terms of temporal criteria and are connected in their historical development) [6,7,8,9,10,11]. The latest definition was proposed late in 2024 by the National Academies of Sciences, Engineering, and Medicine (NASEM); however, none of the included studies has specifically adopted it. This definition was cited only by Mandel et al. [24]. For the present analysis, this study was included among those adopting the National Institute of Health (NIH) definition, as its patient inclusion criteria were consistent with NIH’s definition. (b) Study design (prospective or retrospective cohort studies). (c) Sex. (d) Age class (0–4 years—preschool children; 5–11 years—school-aged children; 12–18 years—adolescents). (e) Prevalent viral strain (pre-Omicron—including studies performed before December 2021; Omicron—including studies performed from December 2021 onwards). (f) Geographic area. Additionally, pooled rates of LC were separately computed for individuals presenting with or without specific clinical conditions, namely, the following: (g) Comorbidities (at least one pre-existing clinical condition, i.e., allergic rhinitis, obesity, previous respiratory diseases; no existing comorbidity during primary SARS-CoV-2 infection). (h) Clinical severity of the primary infection (asymptomatic; mild/moderate or severe SARS-CoV-2 infection). (i) Need for hospital admission during the primary infection (yes/no). (l) Vaccination status (≥1 anti-SARS-CoV-2 vaccine dose or none). Second, we computed the pooled rates of several conditions, all included into the LC-spectrum, selected according to their clinical severity and prevalence [17]: (a) general symptoms (tiredness or fatigue and fever); (b) respiratory symptoms (cough, dyspnea and asthma); (c) musculoskeletal symptoms (myalgia and arthralgya); (d) cardiovascular symptoms; (e) neurological symptoms (headache, dizziness, loss of smell or taste, and concentration and memory problems); (f) gastrointestinal symptoms; (g) dermatological symptoms; and (h) mental health sequelae.

Third, we performed head-to-head meta-analyses to evaluate the likelihood of developing LC in the presence of selected demographic and clinical conditions. In this case, the units of the analyses were subjects with versus subjects without each characteristic, to compute the summary risk of LC among the following: (a) females vs. males; (b) school-age children (6–11 years old) and adolescents (12–18 years old) vs. preschool children (up to 5 years old); (c) subjects with ≥1 comorbidity vs. healthy individuals; (d) subjects with a previous symptomatic COVID-19 vs. those with a previous asymptomatic SARS-CoV-2 infection; (e) individuals requiring hospitalization during the primary infection vs. individuals not requiring hospital admission; and (f) individuals who received ≥1 dose of SARS-CoV-2 vaccine vs. the unvaccinated. For each included study, we extracted only the adjusted estimates of the risk of LC; in case multiple adjusted estimates were available for the same study, the results with the highest level of adjustment were extracted. In case a study reported separate estimates by subgroups, the overall estimate of risk was computed by combining those estimates using the fixed-effect model for generic inverse variance outcomes. Data were then combined using the random-effects generic inverse variance approach, and the results were expressed as summary odds ratios (ORs) and 95% confidence intervals (CIs). Egger’s test was performed to assess publication bias for head-to-head meta-analyses including ≥10 individual studies. The methodological quality of each included study was assessed using the Newcastle–Ottawa Scale for cohort studies [25] and, in order to quantify the role of study quality in affecting the available estimates, when sufficient data were available, sensitivity analyses were performed considering only high-quality studies.

In all analyses, the statistical heterogeneity was quantified using the I^2^ metric. Stata, version 13.1 (StataCorp., College Station, TX, USA, 2014) and RevMan, version 5.4 (CThe Nordic Cochrane Centre, The Cochrane Collaboration, Copenhagen, Denmark, 2020) were used to perform proportion and head-to-head meta-analyses, respectively.

## 3. Results

### 3.1. Characteristics of the Included Studies

**Figure 1:**
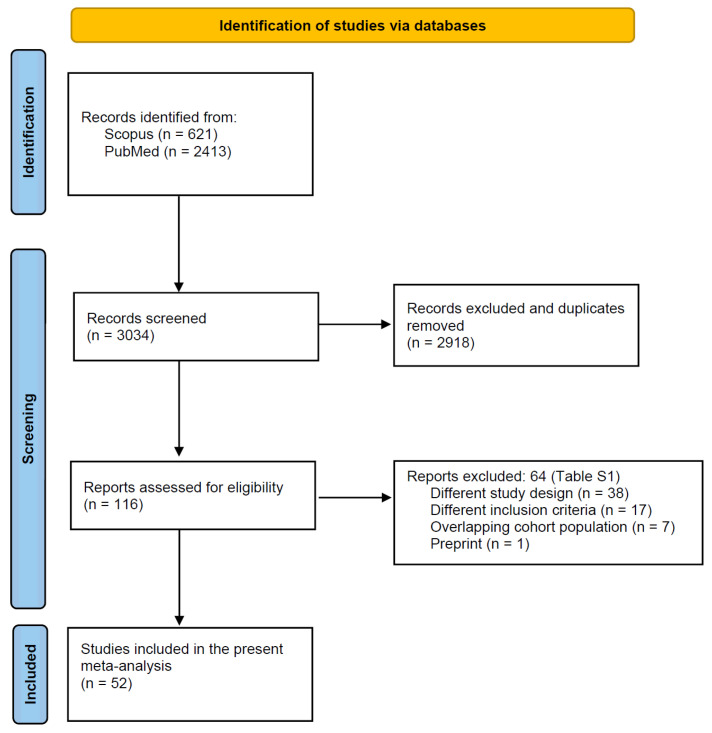
PRISMA 2020 flow diagram.

The initial search identified 3034 papers, retrieved across different datasets (2413 from PubMed; 621 from Scopus). After title/abstract screening and duplicates exclusion, a total of 116 studies were assessed for eligibility; of these, 52 cohort studies (39 prospective and 13 retrospective) and 960,089 children and adolescents were included in the analyses (Figure 1). The complete list of the 64 excluded studies and the corresponding reasons for exclusion are reported in Table S1. The general characteristics of the 52 included publications are summarized in Table 2. The majority of the selected publications (41 out of 52) were performed in Europe and the US. Most studies (*n* = 36; 132,371 participants) enrolled children from unselected, general populations; seven cohorts (*n* = 824,869 participants) analyzed a mixed sample of general population and hospitalized individuals; and nine publications (*n* = 2849 participants) included only hospitalized subjects. Four large international cohorts provided data for ten of the included studies [24,26,27,28,29,30,31,32,33,34]. In particular, (a) two studies [26,27] included hospitalized children from the Children’s Clinical University Hospital in Riga, Latvia; (b) two studies [28,29] referred to a cohort evaluated at the Z.A. Bashlyaeva Children’s Municipal Clinical Hospital in Moscow, Russia; (c) three studies [30,31,32] referred to a large UK-based cohort (CLoCK study) [35]; and (d) three studies [24,33,34] referred to the NIH-founded RECOVER initiative (researching COVID to Enhance Recovery) PEDSnet cohort [6]. Since studies referring to the same cohort were likely to include overlapping populations, we selected, from each cohort, the publication with the longest follow-up, specifically Roge et al. [27] for the Latvian cohort, Pazukhina et al. [29], for the Moscow cohort, Stephenson et al. [32] for the CLOCK study, and Mandel et al. [24] for the RECOVER initiative. The remaining six studies [26,28,30,31,33,34] were included only in the stratified analyses, in case enough data were available.

### 3.2. Pooled Rates of Long-COVID

The overall rate of LC among children and adolescents was 18.1% (95% CI: 13.1–23.6%—Table 3). When the analyses were repeated considering only high-quality studies, the overall estimate was 12.0% (95% CI: 5.9–19.9%). Substantial differences were observed according to the LC definition used: the studies adopting the WHO criteria yielded the highest pooled rates (21.6%; 95% CI: 17.0–26.5%), while the lowest were observed with studies following the NIH definition (16.2%, 95% CI: 2.6–37.9%). In analyses stratified by gender, age class, and clinical status, females (vs. males), adolescents aged 12–18 years (vs. younger subjects), and individuals with a previous severe COVID-19 or requiring hospital admission during the primary infection (vs. asymptomatic individuals) reported a higher burden of LC, with pooled rates ranging from 18.3% to 26.8%. Differences also emerged, considering the viral strain responsible for the primary infection: the pooled rates of LC were as high as 21.6% following Omicron primary infections, as compared to pre-Omicron (16.4%). Finally, when the analyses were stratified by vaccination status, similar rates emerged across groups: the overall estimate was 8.3% (95% CI: 0.9–21.7%) among unvaccinated subjects and 9.3% (95% CI: 2.0–21.1) for those receiving ≥1 vaccine dose (Table 3).

Three of the included studies [24,33,34] adopted a specific time span to define an LC diagnosis (30–180 days following a primary infection), compared with the other included studies, which generally considered more generic time frames (often extending beyond 6 months after primary infection) without a defined upper limit. As such, we carried out sensitivity analyses after the exclusion of these studies, re-computing both overall and stratified pooled rates, with no substantial changes in the observed summary estimates (Table S1). Most cohorts were followed for three to six months after the primary infection, and the corresponding pooled estimates of LC were around 20%. Only four studies (based upon 13,255 subjects) provided data up to 24 months [32,36,37,38] and, after pooling their results, a likely spontaneous improvement of symptoms over time emerged, and the resulting pooled LC estimates were markedly lower (9.2–95% CI: 0.6–25.9%).

**Table 2:** Characteristics of the included studies.

First Author	Journal	Year	Country	Study Design	LC Definition	Total Sample	Population	Mean Age	% Males	Time-Points f-up
Smane [26]	*BMC Pediatr Open*	2020	Latvia	Retr	Own definition	92	general	9.2 (5.2)	56.7	3 m
Blomberg [39]	*Nat Med*	2021	Norway	Pros	Own definition	247	general	8 (6–12)	44.0	6 m
Fink [40]	*Clinics*	2021	Brazil	Pros	NIH	53	general	14.6 (8–18)	57.0	3 m
Matteudi [41]	*Acta Paediatr*	2021	France	Pros	NICE	154	mixed	9.1	NR	12 m
Molteni [42]	*Lancet Child Adolesc Health*	2021	UK	Pros	NICE	1734	general	13 (10–15)	49.8	1 m
Radtke [43]	*JAMA*	2021	Switzerland	Pros	Own definition	1355	general	11 (9–13)	46.0	1 m; 3 m
Roge [27]	*Front Pediatr*	2021	Latvia	Pros	NICE	378	general	10 (5–14)	55.5	1 m
Say [44]	*Lancet Child Adolesc Health*	2021	Australia	Pros	WHO	151	general	3 (1–8)	53.0	3 m
Sterky [45]	*Acta Paediatr*	2021	Sweden	Pros	NICE	55	hospitalized	range 0–18	58.0	6 m
Bergia [46]	*Acta Paediatr*	2022	Spain	Retr	NICE	549	mixed	7 (5.3)	55.0	3 m
Bloise [47]	*Ital J Pediatr*	2022	Italy	Pros	WHO	1412	general	10 (6–13)	51.2	3 m
Borch [48]	*Eur J Pediatr*	2022	Denmark	Pros	NICE	30,121	general	7 (1.5)	NR	1 m
Buonsenso A [49]	*J Clin Med*	2022	Italy	Pros	NICE	679	general	10 (6–13)	49.0	1 m; 6 m; 12 m
Dumont [50]	*Nat Commun*	2022	Switzerland	Pros	CloCK Consortium	1034	general	10.2 (4.2)	51.0	1 m; 3 m
Erol [51]	*Cardiol Young*	2022	Turkey	Retr	WHO	216	general	8.8 (8.7–17.4)	53.7	3 m
Funk [52]	*JAMA Netw Open*	2022	Multicountry	Pros	NIH	1884	mixed	3 (0–10)	52.8	3 m
Güven [53]	*Eur Rev Med Pharmacol Sci*	2022	Turkey	Retr	NICE	502	general	12.6 (4.83)	49.8	1 m
Maddux [54]	*Pediatrics*	2022	US	Pros	NIH	119	hospitalized	NR	49.6	3 m
Messiah [55]	*Ped Infect Dis J*	2022	US	Pros	NICE	1813	general	13.4 (3.7)	50.7	1 m; 3 m
Miller [56]	*Pediatr Infect Dis J*	2022	UK	Pros	NICE	5032	general	range 2–17	44.5	1 m
Osmanov [28]	*Eur Respir J*	2022	Russia	Pros	WHO	518	hospitalized	10.4 (3–15.2)	47.9	6 m
Pazukhina [29]	*BMC Med*	2022	Russia	Pros	WHO	360	hospitalized	9.5 (2.4–14.8)	48.0	12 m
Roessler [57]	*PLoS Med*	2022	Germany	Retr	WHO	57,763	general	range 0–17	51.9	6 m
Trapani [58]	*Ital J Pediatr*	2022	Italy	Pros	NICE	689	general	6 (0–11)	51.9	3 m; 6 m
Zavala [59]	*Clin Infect Dis*	2022	UK	Pros	NICE	474	general	10 (6–13)	49.8	1 m
de Lima [38]	*Pediatr Infect Dis J*	2023	Portugal	Retr	NICE	237	general	6.5	54.4	1 m; 3 m; 6 m
Ertesvåg [60]	*eBioMedicine*	2023	Norway	Pros	WHO	276	general	16.5 (10–20)	46.0	3 m; 6 m
Jarupan [61]	*Vaccines*	2023	Thailand	Pros	NIH	154	general	9 (7–13)	39.0	3 m; 6 m
Körner [62]	*Children*	2023	Germany	Pros	CLoCK Consortium	28	general	13.7 (7.3–17.6)	43.0	3 m
Li [63]	*Ann Acad Med Singap*	2023	Singapore	Retr	CLoCK Consortium	640	general	5.8 (2.0–10.2)	53.5	3 m; 6 m
Mancino [64]	*Int J Environ Res Public Health*	2023	Italy	Pros	WHO	697	general	9.6 (0.1–18.6)	52.2	1 m; 3 m
Pinto Pereira A [31]	*Arch Dis Child*	2023	UK	Pros	CLoCK Consortium	6407	general	range 11–17	37.4	6 m
Pinto Pereira B [30]	*Children*	2023	UK	Pros	CLoCK Consortium	8060	general	range 11–17	37.7	12 m
Sedik [65]	*BMC Pediatr*	2023	Iraq	Pros	CLoCK Consortium	105	mixed	6.3 (4–12)	51.4	1 m; 3 m; 6 m
Seery [66]	*Int J Infect Dis*	2023	Argentina	Pros	CLoCK Consortium	1216	hospitalized	7.5 (5–12)	53.0	3 m
Warren-Gash [36]	*BMC infect Dis*	2023	UK	Pros	CLoCK Consortium	7797	general	NR	48.4	24 m
Boyarchuk [67]	*Front Immunol*	2024	Ukraine	Pros	WHO	190	hospitalized	1.35 (0.6–5.5)	54.2	3 m
Calcaterra [68]	*Ital J Pediatr*	2024	Italy	Pros	WHO	167	hospitalized	range 0–18	53.9	3 m
Camporesi [37]	*EClinicalMedicine*	2024	Italy	Pros	CLoCK COnsortium	1319	general	7.2 (4–10.3)	54.1	3 m; 6 m; 12 m; 24 m
Kostev [69]	*Pediatr Res*	2024	Germany	Retr	NICE	6568	general	10 (5)	50.8	3 m
Sansone [70]	*Pediatr Pulmonol*	2024	Italy	Pros	CloCK Consortium	58	general	10.8 (4.1)	52.0	3 m
Sarani [71]	*BMC infect Dis*	2024	Iran	Retr	WHO	282	mixed	6.6 (0.4)	42.6	3 m
Stephenson [32]	*Commun Med*	2024	UK	Pros	CLoCK Consortium	5177	general	range 11–17	35.5	24 m
Wongwatha- navikrom [72]	*Pediatr pulmonol*	2024	Thailand	Pros	NICE	116	hospitalized	7 (2–12)	52.6	3 m
Britton [73]	*Pediatr Res*	2025	Australia	Pros	CLoCK Consortium	1447	general	NR	43.2	6 m
Dixon [74]	*J Infect Public Health*	2025	US	Retr	NIH	93,901	mixed	7.2 (5.9)	50.4	3 m
Esposito [75]	*Front Immunol*	2025	Italy	Pros	PC-COS	1129	general	7.7 (4.4)	51.9	3 m; 6 m; 12 m
Gross [33]	*JAMA Pediatr*	2025	US	Pros	NIH	1011	general	2.5 (0.83)	49.5	12 m
Iijima [76]	*Pediatr Int*	2025	Japan	Pros	NICE	108	hospitalized	2.4 (0.6–7.2)	55.0	1 m; 3 m; 6 m
Mandel [24]	*Clin Infect Dis*	2025	US	Retr	NIH/NASEM	727,994	mixed	9 (7)	50.0	3 m
Rao [34]	*EClinicalMedicine*	2025	US	Retr	NASEM	203,365	general	NR	50.2	6 m
Yang [77]	*J Med Econ*	2025	France	Retr	NICE	27,537	general	8.9 (5.2)	50.8	12 m

LC: Long-COVID; NR: not reported; Retr: retrospective cohort study; Pros: prospective cohort study. NICE: National Institute for Health and Care Excellence; NIH: National Institute of Health; CLoCK: Children and Young People with Long-COVID; PC-COS: Post-COVID Core Outcome Set; WHO: World Health Organization; NASEM: National Academies of Sciences, Engineering, and Medicine.

**Table 3:** Pooled Rates of Long-COVID among pediatric subjects with a previous history of laboratory-confirmed SARS-CoV-2 infection. Data from single studies have been combined using proportion meta-analysis (random-effects model).

Outcomes	N. Studies	Raw Data (*n*/N)	Pooled Rates % (95% CI)	I^2^, %
**Overall sample ^A^**				
- Overall rate of Long-COVID [24,27,29,32,36,37,38,39,40,41,42,43,44,45,46,47,48,49,50,51,52,53,54,55,56,57,58,59,60,61,62,63,64,65,66,67,68,69,70,71,72,73,74,75,76,77]	46	85,378/960,089	18.1 (13.1–23.6)	99
**Stratified analyses**				
(a) By the adopted Long-COVID definition: ^B^				
- NICE [27,38,45,46,48,49,51,53,55,56,58,59,69,72,76,77]	16	8902/56,994	17.1 (11.3–23.9)	99
- NIH/NASEM [24,34,40,52,54,61]	6	62,573/823,959	16.2 (2.6–37.9)	99
- CLoCK Consortium/PCCOS [32,36,37,50,62,63,65,66,70,73,75]	11	2321/17,824	18.3 (10.1–28.3)	99
- WHO [29,44,47,57,60,64,67,68,71]	10	12,564/61,114	21.6 (17.0–26.5)	95
(b) By study design:				
- Prospective cohort [27,29,32,36,37,39,40,41,42,43,44,45,47,48,49,50,52,54,55,56,58,59,60,61,62,64,65,66,67,68,70,72,73,75,76]	35	7763/44,839	18.6 (13.9–23.7)	99
- Retrospective cohort [24,38,46,51,53,57,63,69,71,74,77]	11	78,615/915,250	16.2 (7.1–28.2)	99
(c) By study quality				
- High-quality studies [22,24,33,36,37,46,47,48,51,58,68,69]	12	41,495/792,275	12.0 (5.9–19.9)	99
- Low- or fair-quality studies [24,26,29,35,38,39,40,41,42,43,44,45,48,50,52,54,55,56,57,59,60,61,62,63,64,65,66,67,70,71,72,73,74]	34	44,883/167,814	20.2 (15.1–25.9)	99
(d) By sex:				
- Females [24,32,36,42,44,45,46,47,49,50,52,53,54,57,58,64,65,67,68,69,70,71,72,74,75,77]	26	42,257/469,991	18.3 (11.6–26.0)	99
- Males [24,32,36,42,44,45,46,47,49,50,52,53,54,57,58,64,65,67,68,69,70,71,72,74,75,77]	26	39,680/466,700	16.6 (10.3–23.9)	99
(e) By age class:				
- 0–4 y [24,41,44,45,48,50,52,54,58,65,67,69,71,73,74,75,76]	17	22,218/308,467	11.6 (4.5–21.3)	99
- 5–11 y [24,36,41,42,44,45,50,52,54,55,58,61,65,67,69,71,73,74,75]	19	17,222/212,592	11.3 (4.3–20.8)	99
- 12–18 y [24,32,36,41,42,44,45,49,50,52,54,55,57,58,60,61,65,68,69,71,73,74,75]	23	26,199/266,789	19.8 (12.3–28.5)	99
(f) By prevalent viral strain:				
- Pre-omicron [27,29,32,38,39,40,41,42,43,44,45,46,47,48,50,51,53,56,57,58,59,61,62,64,65,66,69,73]	30	18,947/98,461	16.4 (12.5–20.6)	99
- Omicron [63,67,74,75,76]	5	33,591/95,681	21.6 (10.5–35.4)	99
(g) By geographical area:				
- Europe [27,32,36,37,38,39,41,42,43,45,46,47,48,49,50,51,53,56,57,58,59,60,62,64,67,68,69,70,75,77]	30	23,344/130,551	19.0 (15.2–23.1)	99
*-* America [24,40,54,55,66,74]	6	62,725/824,331	18.7 (3.8–41.0)	99
- Asia [29,61,63,65,71,72,76]	7	226/1421	18.8 (11.3–27.6)	93
- Oceania [44,73]	2	75/1902	3.8 (3.0–4.7)	-
- Multicountry [52]	1	108/1884	5.7 (4.8–6.9)	-
(h) By clinical status before primary SARS-CoV-2 infection:				
- No comorbidities [24,36,45,46,52,55,65,67,70,71,73,74]	12	38,454/708,123	17.4 (7.7–29.8)	99
- At least one comorbidity [24,36,45,46,52,55,65,67,70,71,73,74]	12	24,920/123,706	24.0 (10.6–40.6)	99
(i) By clinical status due to primary SARS-CoV-2 infection:				
*-* Asymptomatic SARS-CoV-2 infection [41,47,52,70]	4	35/544	5.0 (3.0–7.3)	0
*-* Mild/moderate COVID-19 [41,47,52,55,65,67,70,73]	8	496/3797	21.9 (11.2–34.8)	98
- Severe COVID-19 [52,55,65,67,73]	5	68/425	26.8 (9.7–47.5)	66
(l) By hospitalization status during primary SARS-CoV-2 infection:				
*-* Subjects not requiring hospitalization [24,41,46,52,65,71,74]	7	48,349/601,750	14.8 (2.3–35.1)	99
- Subjects requiring hospitalization [24,41,46,52,65,71,74]	7	14,655/193,386	24.4 (6.2–49.1)	99
(m) By anti-SARS-CoV-2 vaccination status:				
- Unvaccinated subjects [24,31,37,55]	4	24,542/608,426	8.3 (0.9–21.7)	99
- Subjects receiving ≥1 anti-SARS-CoV-2 vaccine dose [24,31,37,55]	4	6594/128,408	9.3 (2.0–21.1)	99

CI: Confidence interval. Raw data show the number of subjects with Long-COVID (*n*) upon the total number of subjects with a previous history of laboratory-confirmed SARS-CoV-2 infection (N). ^A^ By the longest follow-up available. ^B^ See methods and Table 1 for further information about the different LC definitions.

The most commonly symptoms reported by LC subjects were fatigue (33.9%; 95% CI: 24.6–43.7%), respiratory symptoms including cough (25.0%; 95% CI: 15.5–35.8%) and dyspnea (18.8%; 95% CI: 12.7–25.7%), and neurological symptoms (24.2%; 95% CI: 18.9–29.8%), with headache (18.4%; 95% CI: 13.2–24.2%), concentration problems (16.3%; 95% CI: 10.1–23.5%) and loss of taste or smell (15.2%; 95% CI: 9.3–22.2%) showing the highest rates. Mental health sequelae were also common (21.4% of the included subjects were affected), while the pooled rates of cardiovascular symptoms were lower (6.7% of the included subjects affected—Table 4). The exclusion of data from the RECOVER cohort [24,33,34] did not significantly change the pooled estimates (Table S2).

### 3.3. Likelihood of Developing Long-COVID

In total, 24 datasets and 843,980 subjects, from 21 different studies, were included in the head-to-head meta-analyses evaluating the association between selected demographic and clinical conditions and LC onset (Table 5; Figures S1–S7). Females, children aged 6–11 years, and adolescents (as compared to infants), individuals with ≥1 comorbidity, and those with symptomatic COVID-19 and/or requiring a hospital admission during the acute phase of SARS-CoV-2 infection (compared to healthy included subjects) showed a significantly higher likelihood of developing LC (all *p* < 0.05; Table 5). No significant differences in the likelihood of developing the condition emerged between the children/adolescents previously vaccinated with ≥1 anti-SARS-CoV-2 vaccine dose, as compared to the unvaccinated (pooled OR: 0.92; 95% CI: 0.61–1.41; 6 datasets; *n* = 761,181). Again, the exclusion of the RECOVER cohort [24,33,34] did not substantially change the pooled estimates (Tables S3–S5). When the analyses were restricted to the subgroup of studies rated as high-quality, female gender, younger age, and presence of co-morbidities were no longer associated with a significant increase in the likelihood of disease (all *p* > 0.05—Table 5). Conversely, sensitivity analyses confirmed the role of a previous severe COVID-19 as a risk factor for developing LC (pooled OR: 2.01–95% CI: 1.95–2.07), and no significant differences were still registered among vaccinated versus unvaccinated subjects (pooled OR: 0.71; 95% CI: 0.38–1.34—Table 5).

### 3.4. Study Quality

The methodological quality of the 52 included studies was assessed by adopting the Newcastle–Ottawa Scale for cohort studies [25], which is reported in Table S6. Overall, 38 of the 52 studies (73%) included in the proportion meta-analyses, and 15 out of 21 included in the head-to-head meta-analyses (71.5%) were rated as having poor or fair quality. One study (2%) showed a low quality in the selection section, 11 (21%) showed a fair quality, and 40 (77%) showed a good quality. Regarding the comparability section, 33 studies scored 0–1 stars, and 19 were rated two stars. In the outcome section, six studies (12%) showed a low quality, 23 (44%) a fair quality (two stars), and 23 (44%) a high quality (three stars).

### 3.5. Publication Bias

The presence of publication bias was assessed visually through funnel plots, and confirmed through Egger’s regression tests: in each meta-analysis with ≥10 individual studies, no evidence of publication bias emerged (see Figure S8 for funnel plots and Egger’s tests).

## 4. Discussion

The main findings of this meta-analysis, based upon a total of 52 studies and more than 960,000 children and adolescents, are the following: first, in line with most previously published meta-analyses and systematic reviews [2,18,78], approximately one fifth of the pediatric population reporting a primary SARS-CoV-2 infection is likely to develop a subsequent LC, with fatigue, respiratory symptoms and neurological morbidity as predominant clinical features. Second, the present findings confirm the modulating role of some demographic and clinical characteristics. Specifically, female gender, older age, and the presence of ≥1 comorbidity are associated with a significantly higher likelihood of a LC diagnosis. A partially different scenario, instead, arises when considering the role of different viral strains (with Omicron increasing the likelihood of LC, as compared to previous strains), and of anti-SARS-CoV-2 vaccination, which does not appear to affect LC onset. Third, when only high-quality studies were considered, two main pictures emerged: (a) the above findings were strongly confirmed only for COVID-19 severity (still a significant predictor of developing LC), and for vaccination status (which did not appear to significantly influence the likelihood of the disease); (b) the pooled rates of LC decreased, up to 12%. However, any consideration of the influence of study quality on the pooled estimates should be interpreted with caution, as the available high-quality evidence on the topic is almost entirely dominated by a single study [24]. Fourth, the adoption, across the included studies, of different definitions results in widely variable estimates, ranging from 16% to almost 22%, and largely depending upon the diagnostic criteria set by each issuing organization. Additionally, it must be considered that, apart from the international criteria adopted, current clinical definitions of LC are generally broad and unspecific, in order to ensure that as many patients as possible have access to health services [79]. Similar to all the previous systematic reviews already published on the topic [19,78,80,81,82], we also found large discrepancies in case definitions, follow-up length, setting (hospitalized- or community-settings, or both), as well as in the level of accuracy in SARS-CoV-2 identification (to select primary cases and, accordingly, the number of LC patients), and in the precision of clinical assessment of LC symptoms (in-person hospital evaluation or through surveys or interviews). This widely variable scenario, inevitably, adds a further layer of heterogeneity and may severely limit the comparisons between studies [5,83,84].

As mentioned above, the majority of the available literature with mixed children/adolescent-adult populations found that female gender, presence of comorbidities, and older age increase the likelihood of developing LC [85]. Specific evidence on the pediatric population, although still based upon a more limited number of studies, shows similar findings, which are also supported by the present meta-analysis [18,20,78,79,80,86,87]. Several studies on adult populations investigating the association between age and LC described it as a “inverted U-shaped curve”, with a roughly linear increase in the absolute LC risk per decade, a peak among middle-aged adults (40 to 60 years old) and then a progressive decline, up to the age class ≥ 80 years, showing estimates comparable to individuals aged 18–24 years [88,89]. In this scenario, it has been initially inferred that pediatric subjects were among those with the lowest absolute risk, and this advantage was explained (a) with the generally milder course of COVID-19 (a recognized risk factor for LC) among children and adolescents, as compared to older subjects [75], (b) with the senescence and functional decline of the immune system [88]. More recently, however, an alternative hypothesis has suggested that the same reduced immune response typical of older ages could play a protective role against LC, which is, essentially, an immune-mediated phenomenon [90]. Notably, this hypothesis, which may challenge the biological advantage hypothesized for younger individuals, seems to be supported by some of the most recent estimates, as well as the present meta-analysis, showing comparable LC rates among pediatric and adult subjects and ranging, according to the available estimates and adopted criteria, from 10% to 25% [75,91].

Conversely, the available literature on the role of different viral variants and on the vaccination status appears less univocal and more difficult to interpret. Regarding the role of viral strains, COVID-19 severity is a known risk factor for LC development [86,92]. Accordingly, previous summary estimates [93] suggested that the Omicron variant, generally associated with a lower risk of severe disease, hospitalization and death among adults, was responsible for a marked decline in LC prevalence, as compared with Alpha and Delta waves. From our results, instead, LC occurred more frequently during Omicron than during pre-Omicron periods. However, these discrepant findings should be interpreted with caution, given that the meta-analysis mentioned above included only adult individuals, and its pooled estimates for the Alpha-variant were collected only from hospitalized subjects [55].

So far, the available data on the role of COVID-19 vaccination to prevent the onset (or limit the severity) of LC have been sparse, and mostly based upon adult individuals [75]. The results of a previous systematic review [94] suggest mixed, contradictory findings, with roughly half of the ten included studies reporting a protective effect of vaccination, increasing with the number of administered doses, and half showing no role of immunization. Our pooled results, although based on a restricted sub-sample of studies, suggest non-significant differences between vaccinated and unvaccinated subjects. However, the present findings should be interpreted with caution: indeed, several potentially relevant factors, including vaccine type, number of doses, timing of vaccination, prior immunity, circulating SARS-CoV-2 variants, and individual risk profiles, could not be adequately assessed because of limited and heterogeneous reporting across studies. Moreover, no stratified analyses could be performed to assess whether a dose–response effect exists or whether greater protection is achieved after completion of a full immunization cycle. Therefore, the current evidence remains insufficient to draw definitive conclusions regarding the effect of vaccination on LC risk among children, and additional, well-designed prospective studies are needed to clarify this association.

Meta-regression might have been used to explore either the causes of heterogeneity or the independent contribution of each recorded factor in determining the likelihood of LC. However, any model that we fit was at serious risk of bias, mainly because of the relatively scarce number of studies included in each meta-analysis, and, moreover, because almost all studies with a control group were markedly imbalanced between LC-positive and LC-negative subjects (sometimes with 1 positive versus 10 or more negative subjects). Thus, the overall value of a given potential risk factor (such as age) of an unbalanced study was very similar to the mean age of the LC-negative group, while studies with more balanced groups seemed to have a higher mean age than the unbalanced, regardless of their relative risk of the outcome. Even exploring the possibility of alternative options (such as age differential between study groups), the bias caused by the scarcity of balanced-group studies severely limited the reliability of any meta-regression.

In this complex and multifaceted scenario, several knowledge gaps still exist, which limit the generalizability of the present findings: first, with most studies providing data from European and North-American subjects, assessing the impact of LC in other geographical areas, and among different ethnic groups is even more difficult. Second, the lack of a standardized definition and of uniform data collection processes to identify LC-related signs and symptoms (which are collected through self-administered questionnaires, telephone interviews, caregiver-reported surveys and clinical examinations) limits the possibility of solid comparisons across all the available estimates, as it influences the completeness of symptoms reporting and, consequently, the pooled LC estimates. Unfortunately, only a fraction of the studies included in the present meta-analyses explicitly mentioned the adopted methodology, and almost entirely coincided with the studies rated as having a high quality. Thus, if a more uniform diagnostic approach is certainly required to ease the comparability across the available estimates, the present findings, based upon high-quality studies, add to the existing literature, providing solid and highly standardized data. Additionally, the adoption, across the available literature, of follow-up length widely varying (in our search, only one study set precise temporal limits within which to identify possible LC cases) inevitably contributes to the observed variation in the reported estimates. Finally, it should be acknowledged that other factors, such as socio-economic status, ease of access to healthcare services, and quality of testing strategies, may have influenced the likelihood of developing (and accurately diagnosing) LC [95]. However, insufficient information was available from the included literature; thus, their specific role could not be adequately addressed.

As a side note, the present meta-analysis was not prospectively registered, as recommended by PRISMA guidelines to ensure data transparency, reproducibility, and methodological rigour [22]. However, building upon several previously published meta-analyses on the same topic, it updates the available evidence on a highly standardized area of research, and, with the specific objective to ease the potential comparability between the present results and those previously published, we strictly followed the methodological and clinical criteria already adopted by previous meta-analyses.

In conclusion, the present findings suggest that, although the available estimates may largely vary depending on the adopted definition, LC appears to affect a meaningful proportion of the pediatric population previously infected with SARS-COV-2. As such, there is an urgent need to unify both clinical criteria and diagnostic approaches. Moreover, the current evidence remains limited by substantial methodological heterogeneity and the generally poor-to-fair quality of a large portion of the available literature. Thus, high-quality studies, performed with standardized criteria, are required to identify at-risk populations in order to support the early recognition of LC and guide the implementation of targeted follow-up strategies.

## Figures and Tables

**Table 1:** List of the different definitions of Long-COVID reported in the available literature on the topic.

Organization/ Institution	Year	Nomenclature	Definition of Pediatric Long-COVID	Functional Impairment, Definition and Tools
NICE ^A^ [7]	2021	(a) Ongoing symptomatic COVID-19 (b) Post-COVID-19 syndrome	(a) Symptoms lasting 4 to 12 weeks after a confirmed SARS-CoV-2 infection; (b) Symptoms lasting >12 weeks after a confirmed SARS-CoV-2 infection	Tools adopted to score not mentioned. Generally defined as “Increased absence or reduced performance in education, work or training”.
NIH ^B^ [6]	2022	Post-acute sequelae of SARS-CoV-2 infection or PASC	Ongoing, relapsing or development of symptoms, or other health effects, occurring after the acute phase of SARS-CoV-2 infection, presenting ≥4 weeks after the acute infection	Several domains assessed through multiple standardized scales, evaluating: full body physical functioning; work-related functioning; activities of daily living; pain; fatigue; cognitive dysfunction. (https://www.nationalacademies.org/read/27756/chapter/6#164 (accessed on 3 July 2026)
CLoCk ^C^ Consortium [8]/PC-COS ^D^ Children Study Group [10]	2022 / 2024	Post-COVID-19 condition or PCC	Among young people with a history of confirmed SARS-CoV-2 infection, persistence of one or more physical symptoms for a minimum of 12 weeks after the initial test, which cannot be explained by an alternative diagnosis.	Core Outcomes Measures Set (COMS) identified through consensus, assessing: gastrointestinal symptoms; fatigue/exhaustion; neurocognitive impairment (all measured through PedsQL scales), plus physical functioning (EQ-5D).
WHO ^E^ [11]	2023	Post-COVID-19 condition or PCC	Symptoms lasting ≥2 months that usually begin within 3 months of the confirmed or probable SARS-CoV-2 infection	Different tools adopted, not mentioned. Main features included changes in eating habits, physical activity, behaviours, educational performance, social functions.
NASEM ^F^ [9]	2024	Long-COVID or LC	SARS-CoV-2 infection-associated chronic condition that occurs and persists ≥3 months, with a disease course that may be continuous, relapsing, remitting, or progressive, and that affects one or more organ systems	Tools adopted to score not mentioned. Generally defined as “Impairment in work, school, ability in taking care of themselves and others. It can have a profound emotional and physical impact on patients and their families and caregivers”.

^A^ National Institute for Health and Care Excellence; ^B^ National Institute of Health; ^C^ Children and Young People with Long-COVID; ^D^ Post-COVID Core Outcome Set; ^E^ World Health Organization; ^F^ National Academies of Sciences, Engineering, and Medicine.

**Table 4:** Pooled rates of each symptom among pediatric subjects with a diagnosis of Long-COVID, by the longest follow-up available. Data from single studies have been combined using proportion meta-analysis (random-effect model).

Outcomes	N. Studies	Raw Data (*n*/N ^A^)	Pooled Rates % (95% CI)	I^2^, %
(a) General symptoms [27,29,33,37,38,40,44,45,46,47,49,50,52,54,58,59,60,61,62,63,64,65,66,67,68,70,71,72,75,76]	30	715/3304	33.8 (24.7–43.5)	96
- Fever [27,33,37,46,50,52,54,59,60,64,67]	11	114/738	11.0 (2.6–23.6)	94
- Fatigue [27,29,37,38,40,44,45,46,47,49,50,52,54,58,60,61,62,63,64,65,66,67,68,70,71,72,75,76]	28	681/3219	33.9 (24.6–43.7)	96
(b) Respiratory symptoms [27,29,34,37,38,40,44,45,46,47,49,50,52,54,58,59,60,61,62,63,64,66,67,68,70,71,72,75,76]	29	3761/14,197	25.8 (19.4–32.7)	96
- Cough [27,28,33,37,38,44,46,49,50,52,54,59,61,62,63,64,66,68,70,71,72,76]	22	356/1665	25.0 (15.5–35.8)	95
- Dyspnea [28,33,37,38,40,46,47,50,52,54,60,61,62,63,64,66,70,72]	18	219/1385	18.8 (12.7–25.7)	88
- Asthma [37,52,64]	3	16/236	6.6 (3.6–10.3)	0
(c) Musculoskeletal symptoms [27,29,37,40,46,47,49,50,54,58,59,60,61,62,63,64,65,66,67,68,70,72,75]	23	268/3014	11.5 (7.2–16.5)	91
- Myalgia [26,40,45,53,70,72,76]	7	27/197	12.7 (7.7–18.6)	12
- Arthralgia [40,70,72]	3	11/86	10.4 (1.9–23.1)	51
(d) Cardiovascular symptoms [27,29,37,46,47,50,52,58,60,62,63,64,66,67,72,75]	16	96/2601	5.7 (3.1–8.9)	85
(e) Neurological symptoms [27,29,34,37,38,40,43,44,46,47,49,50,52,53,54,58,59,60,61,62,63,64,65,66,67,68,70,71,72,75]	30	2620/14,214	24.2 (18.9–29.8)	94
- Memory problems [27,33,40,59,63,64,66,72]	8	69/674	9.6 (6.4–13.2)	43
- Concentration problems [27,38,40,43,46,47,49,50,61,62,63,64,65,66,71,76]	16	220/1449	16.3 (10.1–23.5)	88
- Dizziness [27,28,46,47,50,52,59,60,62,63,66,72]	12	101/1206	9.7 (5.7–14.5)	82
- Headache [27,28,37,38,40,46,47,49,50,52,53,54,59,60,61,62,63,64,65,66,68,70,71,72,76]	25	349/1959	18.4 (13.2–24.2)	88
- Loss of taste/smell [27,28,37,38,45,46,47,49,50,52,54,58,59,60,62,63,64,66,71,72]	20	272/1969	15.2 (9.3–22.2)	92
(f) Gastrointestinal symptoms [27,29,33,37,43,45,46,47,49,50,52,53,54,58,59,60,61,62,63,64,65,66,67,68,71,72,75,76]	28	292/3257	13.3 (9.1–18.1)	90
(g) Dermatological symptoms [29,33,37,50,58,61,63,64,67,72,75]	11	64/1851	6.8 (3.0–11.8)	88
(h) Mental health sequelae [27,29,33,38,40,43,45,46,47,50,58,59,60,61,62,63,64,66,68,71,72,75,76]	23	357/2677	21.4 (13.8–30.0)	94

CI: Confidence interval. *n*/N ^A^: number of subjects with any specific symptom/total number of subjects with Long-COVID.

**Table 5:** Results of the head-to-head meta-analyses showing the association between each demographic and clinical characteristic and the likelihood of developing Long-COVID. Data from single datasets have been combined using a random-effect model.

Outcomes	N. Datasets (Sample)	Pooled OR (95% CI)	*p*	I^2^, %
(a) Gender, females vs. males [24,29,37,50,52,58,60,63,67,69,73,74,75,76]				
All studies	17 (833,157)	1.08 (1.00–1.16)	0.04	58
High-quality studies [24,52,63]	3 (730,349)	1.05 (0.73–1.50)	0.8	73
(b) Age class:				
All studies				
- 0–5 y [24,28,37,52,58,69,71,73,74,75]	--	1 (ref. cat.)	--	--
- 6–11 y [24,28,37,52,58,69,71,73,75]	9 (446,904)	1.35 (1.04–1.75)	0.02	58
- 12–18 y [28,37,52,58,69,71,73,74,75]	12 (73,737)	1.75 (1.38–2.23)	<0.001	92
High-quality studies [24,52,71]				
- 0–5 y	--	1 (ref. cat.)	--	--
- 6–11 y	3 (439, 758)	1.32 (0.77–2.26)	0.3	61
(c) Presence of comorbidities, yes vs. no				
All studies [24,29,37,50,52,54,55,58,63,67,69,72,73,74]	17 (824,956)	1.68 (1.42–1.98)	<0.001	95
High-quality studies [24,52,55,63,72]	5 (729,436)	1.25 (0.90–1.75)	0.2	57
(d) Symptomatic SARS-CoV-2 infection vs. asymptomatic				
All studies [29,37,41,52,54,58,60,76]	8 (4892)	2.47 (1.37–4.46)	<0.01	88
(e) Severe COVID-19 ^A^, yes vs. no				
All studies [24,37,41,46,52,54,58,63,74]	12 (822,591)	1.88 (1.55–2.27)	<0.001	84
High-quality studies [24,52,63]	3 (730,349)	2.01 (1.95–2.07)	<0.001	0
(f) SARS-CoV-2 vaccinated individuals, vs. unvaccinated ^B^				
All studies [24,37,55,60,63,76]	6 (761,181)	0.92 (0.61–1.41)	0.7	60
High-quality studies [24,55,63]	3 (758,838)	0.71 (0.38–1.34)	0.3	82

OR: odds ratio; CI: confidence interval. ^A^ Severe COVID-19 is a symptomatic disease requiring hospital admission during primary infection. ^B^ At least 1 vaccine dose, vs. none.

## Data Availability

All data are available from the studies that have been included in the meta-analysis.

## Supplementary Materials

The following supporting information can be downloaded at https://www.mdpi.com/article/10.3390/jcm15145597/s1. Table S1: List of the 64 excluded studies after full text screening. Table S2: Overview of the cohorts included in the present meta-analysis. Table S3: Pooled Rates of Long-COVID among pediatric subjects with a previous history of laboratory-confirmed SARS-CoV-2 infection, after excluding the RECOVER cohort. Table S4: Pooled rates of each symptom among pediatric subjects with a diagnosis of long-COVID, by the longest follow-up available, after excluding the RECOVER cohort. Table S5: Results of the head-to-head meta-analyses showing the association between each demographic and clinical characteristic and the likelihood of developing Long-COVID, after excluding the RECOVER cohort. Table S6: Quality evaluation of the included studies, according to the Newcastle–Ottawa Scale for cohort studies. Table S7. PRISMA 2020 Checklist. Figure S1: Likelihood of developing Long-COVID in female versus male subjects with a previous history of laboratory-confirmed SARS-CoV-2 infection. Figure S2: Likelihood of developing Long-COVID in children (5–12 years old) versus infants (<5 years old) with a previous history of laboratory-confirmed SARS-CoV-2 infection. Figure S3: Likelihood of developing Long-COVID in adolescents (12 years old) versus infants (<5 years old) with a previous history of laboratory-confirmed SARS-CoV-2 infection. Figure S4: Likelihood of developing Long-COVID among SARS-CoV-2 positive subjects with at least one comorbidity, versus subjects without a history of comorbidities. Figure S5: Likelihood of developing Long-COVID among subjects with a previous history of symptomatic COVID-19 versus subjects with a previous asymptomatic SARS-CoV-2 infection. Figure S6: Likelihood of developing Long-COVID among subjects with a severe COVID-19 requiring hospitalization versus subjects not requiring hospital admission during the primary infection. Figure S7: Likelihood of developing Long-COVID among subjects receiving ≥1 anti-SARS-CoV-2 vaccine dose, versus unvaccinated individuals. Figure S8: Publication bias assessment; funnel plots and Egger’s test for head-to-head meta-analyses including ≥10 individual studies.

## Abbreviations

The following abbreviations are used in this manuscript:

CARES	Coronavirus Antibody REsponse Survey
CIs	Confidence Intervals
CLOCK	Children and Young People with Long-COVID
COVID-19	CoronaVirus-19-Related Disease
HtH	Head-to-Head Meta-Analysis
LC	Long-COVID
MaP	Meta-Analysis of Proportions
MaPS	Stratified Meta-Analysis of Proportions
NASEM	National Academies of Sciences, Engineering, and Medicine
NCCHD	National Center for Child Health Development
NICE	National Institute for Health and Care Excellence
NIH	National Institute of Health
NR	Not Reported
OR	Odds Ratio
PASC	Post-Acute Sequelae of SARS-CoV-2
PCCs	Post-COVID-19 Conditions
PC-COS	Post-COVID Core Outcome Set
PRISMA	Preferred Reporting Items for Systematic Reviews and Meta-Analyses
RECOVER	Researching COVID to Enhance Recovery
SARS	Severe Acute Respiratory Syndrome
SD	Standard Deviation
THIN	The Health Improvement Network
WHO	World Health Organization
