# Supplementary material for: The Burden of Long-COVID-19 Among Pediatric Subjects: A Systematic Review and Meta-Analysis
Source: J Clin Med. 2026 Jul 16;15(14):5597. doi: 10.3390/jcm15145597 (PMC13413068; doi:10.3390/jcm15145597)
Supplement: Supplementary file 1 [file jcm-15-05597-s001.zip › jcm-4343826-Supplementary material.pdf]

Sequelae of COVID-19 in Hospitalized Children: A 4-Months Follow-Up Supplementary Material

Table S1. List of the 64 excluded studies after full text screening.

First author	Journal	Year	Reason(s) for exclusion
Denina (1)	Pediatr Infect Dis J	2020	different study design
Asadi-Pooya (2)	World J Pediatr	2021	different study design
Ashkenazi-Hoffnung (3)	Pediatr Infect Dis J	2021	different inclusion criteria
Brackel (4)	Pediatr Pulmonol	2021	different inclusion criteria
Buonsenso (5)	Acta Paediatr	2021	different study design
Di Sante (6)	medRxiv	2021	Never published, only preprint
Leftin Dobkin (7)	Pediatr Pulmonol	2021	different inclusion criteria
Ludvigsson (8)	Acta Paediatr	2021	different study design
Nogueira Lopez (9)	Acta Paediatr	2021	different study design
Rusetsky (10)	Laringoscope	2021	different study design
Smane (11)	Ital J Pediatr	2021	same sample of Roge 2021 (12) and different study design
Bode (13)	Front Pediatr	2022	different study design
Buonsenso B (14)	Front Pediatr	2022	same sample of Buonsenso 2022 (15), shorter follow-up
Buonsenso C (16)	Future Microbiol	2022	different inclusion criteria
Di Gennaro (17)	Sci Rep	2022	different study design
Dolezalova (18)	Acta Paediatr	2022	different inclusion criteria
Gonzalez-Aumatell (19)	Children	2022	different inclusion criteria
Guido (20)	Front Neurol	2022	different study design
Kikkenborg Berg A (21)	Lancet Child Adolesc Health	2022	different study design
Kikkenborg Berg B (22)	Lancet Child Adolesc Health	2022	different study design
Stephenson (23)	Lancet Child Adolesc Health	2022	different inclusion criteria
Adler (24)	BMJ Open	2023	different study design
Ahn (25)	Pediatr Infect Dis J	2023	different study design
Al-Shamrani (26)	Children	2023	different study design
Atchison (27)	Arch Dis Child	2023	different study design
Balderas (28)	Front Pediatr	2023	different inclusion criteria
Buonsenso (29)	J Clin Med	2023	different study design
Garai (30)	Pediatr Res	2023	different inclusion criteria

(continued)

First author	Journal	Year	Reason(s) for exclusion
Heiss (31)	Radiology	2023	different study design
Morello (32)	EClinicalMedicine	2023	same sample of Camporesi 2024 (33), shorter follow-up.
Paniskaki (34)	Pediatr Allergy Immunol	2023	different study design
Shmueli (35)	J Clin Med	2023	different inclusion criteria
Stephenson (36)	PLoS One	2023	same sample of Pinto Pereira A 2023 (37)
Valenzuela (38)	Pediatr Obes	2023	different inclusion criteria
Delogu (39)	Eur J Pediatr	2024	different inclusion criteria
Foret-Bruno (40)	Brain Behav Immun	2024	same follow-up interval, shorter sample of Stephenson 2023 (41)
Hersh (42)	Child Adolesc Psychiatry Ment Health	2024	different inclusion criteria
Hosozawa (43)	Int J of Infect Dis	2024	different study design
Korkmaz (44)	J Infect Dev Ctries	2024	different inclusion criteria
Mizrahi (45)	BMJ	2024	different inclusion criteria
Motilal (46)	Cureus	2024	different study design
Pazukhina (47)	BMC Med	2024	different inclusion criteria
Pinto Pereira (48)	Sci Rep	2024	same follow-up, smaller sample than Pinto Pereira B 2023 (49)
Razzaghi (50)	Pediatrics	2024	same sample of Mandel 2025 (51)
Schiavo (52)	Children	2024	different study design
Setiabudi (53)	Clin Med Res	2024	different study design
Seylanova (54)	Eur Resp J	2024	different study design
Wang (55)	BMC Public Health	2024	different study design
Chepo (56)	BMC Public Health	2025	different study design
Cianciulli (57)	Infect Dis Rep	2025	different study design
Coughtrey (58)	Curr Opin Infect Dis	2025	different study design
Ford (59)	JAMA Pediatr	2025	different study design
Groohi-Sardou (60)	Curr Pediatr Rev	2025	different study design
Gross (61)	JAMA Pediatr	2025	different study design
Gupte (62)	Pediatr Ann	2025	different study design
Hussein (63)	J Infect Dev Ctries	2025	different study design
Lorman (64)	PLOS Digit Health	2025	different inclusion criteria
Noij (65)	Commun Med	2025	different study design

(continued)

(continued)

First author	Journal	Year	Reason(s) for exclusion
Rojas (66)	J Med Internet Res	2025	different study design
Terry (67)	BMJ Open Respir Res	2025	different study design
Var (68)	J Clin Med	2025	different study design
Wee (69)	Clin Microbiol Infect	2025	different inclusion criteria
Willis (70)	J Pediatr Psychol	2025	different study design
Wurm (71)	J Allergy Clin Immunol	2025	different study design

Table S2. Overview of the cohorts included in the present meta-analysis.

First author	Journal	Sample	Cohort name	Time line	Analyses
				Apr 2020 Oct 2020 Apr 2021 Oct 2021 Apr 2022 Oct 2022 Apr 2023 Oct 2023 Apr 2024	
Smane (72)	BMC Pediatr Open	92	Latvia, Riga 06/2020 - 04/2021		**MaPS**: c.
Roge (12)	Front Pediatr	378	Latvia, Riga, 06/2020 - 04/2021		**MaP**: O, a, b, f, g; **MaPS**: a; b; d; e; f; h.
Osmanov (73)	Eur Respir J	518	Moscow cohort 04/2020-08/2020		**MaPS**: b, e; **HtH**: b1, b2
Pazukhina (74)	BMC Med	360	Moscow cohort 04/2020-08/2020		**MaP**: O, a, b, c, f, g; **MaPS**: a, c, d, f, g ,h; **HtH**: a, c, d
Pinto Pereira A (37)	Arch Dis Child	6407	CLoCK Study 09/2020-03/2021		**MaPS**: d, f.
Pinto Pereira B (49)	Children	8060	CLoCK Study 09/2020-03/2021		**MaP**: m; **MaPS**: a, b, e.
Stephenson (75)	Commun Med	5177	CLoCK Study 09/2020-03/2021		**MaP**: O, a, b, c, d, e, f, g.
Gross (76)	JAMA Pediatr	1011	RECOVER 03/2022-07/2024		**MaPS**: a, f, g, h
Mandel (51)	Clin Infect Dis	727,994	RECOVER 03/2020-02/2023		**MaP**: O, a, b, c, d, f, g, h, l, m; **HtH**: a, b1, c, e, f.
Rao (77)	E Clinical Medicine	203,365	RECOVER 03/2020-12/2022		**MaPS**: b, e.

MaP: meta-analyses of proportions: (O) overall sample and stratified by: (a) adopted Long-COVID definition; (b) study design; (c) study quality; (d) sex; (e) age class; (f) prevalent viral strain; (g) geographical area; (h) clinical status before primary SARS-CoV-2 infection; (i) clinical status due to primary SARS-CoV-2 infection; (l) hospitalization status during primary SARS-CoV-2 infection; (m) anti-SARS-CoV-2 vaccination status.
MaPS: meta-analyses of proportions, pooled rates of symptoms stratified by: (a) general symptoms (tiredness or fatigue and fever); (b) respiratory symptoms (cough, dyspnea and asthma); (c) musculoskeletal symptoms (myalgia and artralgya); (d) cardiovascular symptoms; (e) neurological symptoms (headache, dizziness, loss of smell or taste, and concentration and memory problems); (f) gastrointestinal symptoms; (g) dermatological symptoms; and (h) mental health sequelae.
HtH: head-to-head meta-analyses , units of the analyses were: (a) females vs. males; (b1) school-age children (6-11 years old) and (b2) adolescents (12-18 years old) vs. preschool children (up to 5 years old); (c) subjects with ≥1 comorbidity vs. healthy individuals; (d) subjects with a previous symptomatic COVID-19 vs. those with a previous asymptomatic SARS-CoV-2 infection; (e) individuals requiring hospitalization during the primary infection vs. individuals not requiring hospital admission; and (f) individuals who received ≥1 dose of SARS-CoV-2 vaccine vs. the unvaccinated.

Table S3. Pooled Rates of Long-COVID among pediatric subjects with a previous history of laboratory-confirmed SARS-CoV-2 infection. Data from single studies have been combined using proportion meta-analysis (random-effects model). Overall and stratified analyses were computed after excluding studies that analyzed the RECOVER cohort; see the main text for further details.

Outcomes	N. studies	Raw data (n/N)	Pooled rates % (95% CI)	I^2, %
**Overall sample** [A]				
- Overall rate of Long-COVID, excluding RECOVER cohort (12, 15, 33, 74, 75, 78-117)	45	56,258 / 232,095	18.3 (14.6-22.4)	99
**Stratified analyses**				
(a) By adopted Long-COVID definition:				
- NICE (12, 15, 84, 85, 87, 89, 91, 93, 94, 96-98, 109, 112, 116, 117)	16	8902 / 56,994	17.1(11.3-23.9)	99
- NIH / NASEM, excluding RECOVER cohort (77, 79, 90, 92, 100)	5	36,453 / 95,965	19.6 (4.8-41.0)	99
- CLoCK Consortium / PCCOS (33, 75, 88, 101, 102, 104-106, 110, 113, 115)	11	2321 / 17,824	18.3 (10.1-28.3)	99
- WHO (74, 83, 86, 95, 99, 103, 107, 108, 111)	10	12,564 / 61,114	21.6 (17.0-26.5)	95
(b) By study design:				
- Prospective cohort (12, 15, 33, 74, 75, 78-84, 86-88, 90, 92-94, 96, 97, 99-101, 103-108, 110, 112, 113, 115, 116)	35	7763 / 44,839	18.6 (13.9-23.7)	99
- Retrospective cohort, excluding RECOVER cohort (85, 89, 91, 95, 98, 102, 109, 111, 114, 117)	10	49,495 / 187,256	17.8 (10.8-26.0)	99
(c) By sex:				
- Females, excluding RECOVER cohort (15, 75, 81, 83-86, 88, 90-92, 95, 96, 103, 104, 106-112, 114, 115, 117)	25	26,728 / 103,748	18.8 (13.7-24.5)	99
- Males, excluding RECOVER cohort (15, 75, 81, 83-86, 88, 90-92, 95, 96, 103, 104, 106-112, 114, 115, 117)	25	25,747 / 104,949	17.1 (12.2-22.6)	99
(d) By age class:				
- 0-4y, excluding RECOVER cohort (80, 83, 84, 87, 88, 90, 92, 96, 104, 107, 109, 111, 113-116)	16	12,589 / 44,917	12.3 (5.5-21.3)	99
- 5-11y, excluding RECOVER cohort (80, 81, 83, 84, 88, 90, 92, 93, 96, 100, 104, 106, 107, 109, 111, 113-115)	18	10,324 / 37,468	11.9 (3.6-23.6)	99
- 12-18y, excluding RECOVER cohort (15, 75, 80, 81, 83, 84, 88, 90, 92, 93, 95, 96, 99, 100, 104, 106, 108, 109, 111, 113-115)	22	17,683 / 70,728	20.7 (13.7-28.6)	99
(e) By prevalent viral strain:				
- Pre-omicron (12, 74, 75, 78-89, 91, 94-98, 100, 101, 103-105, 109, 113)	30	18,947 / 98,461	16.4 (12.5-20.6)	99
- Omicron (102, 107, 114-116)	5	33,591 / 95,681	21.6 (10.5-35.4)	99

(continued)

(continued)

Outcomes	N. studies	Raw data (n/N)	Pooled rates % (95% CI)	I^2, %
(f) By geographical area:				
- Europe (12, 15, 33, 75, 78, 80-82, 84-89, 91, 94-99, 101, 103, 106-110, 115, 117)	30	23,344 / 130,551	19.0 (15.2-23.1)	99
- America, excluding RECOVER cohort (79, 92, 93, 105, 114)	5	33,605 / 96,337	22.8 (6.6-44.9)	99
- Asia (74, 100, 102, 104, 111, 112, 116)	7	226 / 1421	18.8 (11.3-27.6)	93
- Oceania (83, 113)	2	75 / 1902	3.8 (3.0-4.7)	-
- Multicountry (90)	1	108 / 1884	5.7 (4.8-6.9)	-
(g) By clinical status before primary SARS-CoV-2 infection:				
- No comorbidities, excluding RECOVER cohort (84, 85, 90, 93, 104, 106, 107, 110, 111, 113, 114)	11	16,266 / 63,252	19.2 (8.5-32.8)	99
- At least one comorbidity, excluding RECOVER cohort (84, 85, 90, 93, 104, 106, 107, 110, 111, 113, 114)	11	17,646 / 40,583	25.8 (10.5-44.8)	99
(h) By clinical status due to primary SARS-CoV-2 infection:				
- Asymptomatic SARS-CoV-2 infection (80, 86, 90, 110)	4	35 / 544	5.0 (3.0-7.3)	0
- Mild/moderate COVID-19 (80, 86, 90, 93, 104, 107, 110, 113)	8	496 / 3797	21.9 (11.2-34.8)	98
- Severe COVID-19 (90, 93, 104, 107, 113)	5	68 / 425	26.8 (9.7-47.5)	66
(i) By hospitalization status during primary SARS-CoV-2 infection:				
- Subjects not requiring hospitalization, excluding RECOVER cohort (80, 85, 90, 104, 111, 114)	6	32,294 / 93,731	17.5 (4.6-36.2)	99
- Subjects requiring hospitalization, excluding RECOVER cohort (80, 85, 90, 104, 111, 114)	6	1248 / 2873	28.3 (9.1-52.7)	98
(l) By anti-SARS-CoV-2 vaccination status:				
- Unvaccinated subjects, excluding RECOVER cohort (33, 37, 93)	3	1487 / 7545	10.1 (0.9-27.3)	99
- Subjects receiving ≥1 anti-SARS-CoV-2 vaccine dose, excluding RECOVER cohort (33, 37, 93)	3	187 / 1295	11.1 (0.1-36.7)	99

CI: Confidence interval. Raw data show the number of subjects with long-COVID (n) upon the total number of subjects with a previous history of laboratory-confirmed SARS-CoV-2 infection (N).

[A] By the longest follow-up available

[B] According to NICE "ongoing symptomatic Covid-19" and NIH definitions.

[C] According to WHO, NASEM, NICE, CLoCK Consortium / PC-COS Children definitions - see methods and Box 1 for further information about the different LC definitions.

Table S4. Pooled rates of each symptom among pediatric subjects with a diagnosis of long-COVID, by the longest follow-up available. Data from single studies have been combined using proportion meta-analysis (random-effects model). Overall and stratified analyses were computed after excluding studies that analyzed the RECOVER cohort; see the main text for further details.

Outcomes	**N. studies**	**Raw data (n/N^A)**	**Pooled rates % (95% CI)**	**I^2, %**
(a) *General symptoms*, *excluding RECOVER cohort* (12, 15, 33, 74, 79, 83-86, 88, 90, 92, 96-105, 107, 108, 110-112, 115, 116)	29	695 / 3243	33.9 (24.5-43.8)	96
- Fever, excluding RECOVER cohort (12, 33, 85, 88, 90, 92, 97, 99, 103, 107)	10	104 / 677	10.5 (1.8-24.4)	95
- Fatigue (12, 15, 33, 74, 79, 83-86, 88, 90, 92, 96, 98-105, 107, 108, 110-112, 115, 116)	28	681 / 3219	33.9 (24.6-43.7)	96
(b) *Respiratory symptoms*, *excluding RECOVER cohort* (12, 15, 33, 74, 79, 83-86, 88, 90, 92, 96-103, 105, 107, 108, 110-112, 115, 116)	28	550 / 3228	25.8 (18.6-33.6)	94
- Cough, excluding RECOVER cohort (12, 15, 33, 73, 83, 85, 88, 90, 92, 97, 98, 100-103, 105, 108, 110-112, 116)	21	311 / 1604	22.7 (14.1-32.6)	94
- Dyspnea, excluding RECOVER cohort (33, 73, 79, 85, 86, 88, 90, 92, 98-103, 105, 110, 112)	17	207 / 1324	18.8 (12.4-26.0)	88
- Asthma (33, 90, 103)	3	16 / 236	6.6 (3.6-10.3)	0
(c) *Musculoskeletal symptoms* (12, 15, 33, 74, 79, 85, 86, 88, 92, 96, 97, 99-105, 107, 108, 110, 112, 115)	23	268 / 3014	11.5 (7.2-16.5)	91
- Myalgia (72, 79, 84, 91, 110, 112, 116)	7	27 / 197	12.7 (7.7-18.6)	12
- Arthralgia (79, 110, 112)	3	11 / 86	10.4 (1.9-23.1)	51
(d) Cardiovascular symptoms (12, 33, 74, 85, 86, 88, 90, 96, 99, 101-103, 105, 107, 112, 115)	16	96 / 2601	5.7 (3.1-8.9)	85
(e) *Neurological symptoms, excluding RECOVER cohort* (12, 15, 33, 74, 79, 82, 83, 85, 86, 88, 90-92, 96-105, 107, 108, 110-112, 115)	29	542 / 3245	24.8 (17.7-32.6)	94
- Memory problems, excluding RECOVER cohort (12, 79, 97, 102, 103, 105, 112)	7	60 / 613	8.9 (5.6-12.8)	44
- Concentration problems (12, 15, 79, 82, 85, 86, 88, 98, 100-105, 111, 116)	16	220 / 1449	16.3 (10.1-23.5)	88
- Dizziness (12, 73, 85, 86, 88, 90, 97, 99, 101, 102, 105, 112)	12	101 / 1206	9.7 (5.7-14.5)	82
- Headache (12, 15, 33, 73, 79, 85, 86, 88, 90-92, 97-105, 108, 110-112, 116)	25	349 / 1959	18.4 (13.2-24.2)	88
- Loss of taste/smell (12, 15, 33, 73, 84-86, 88, 90, 92, 96-99, 101-103, 105, 111, 112)	20	272 / 1969	15.2 (9.3-22.2)	92
(f) Gastrointestinal symptoms, excluding RECOVER cohort (12, 15, 33, 74, 82, 84-86, 88, 90-92, 96, 99-105, 107, 108, 111, 112, 115, 116)	27	278 / 3196	12.9 (8.7-17.8)	90
(g) Dermatological symptoms, excluding RECOVER cohort (33, 74, 88, 96, 100, 102, 103, 107, 112, 115)	10	55 / 1790	6.1 (2.5-10.9)	87
(h) Mental health sequelae, excluding RECOVER cohort (12, 74, 79, 82, 84-86, 88, 96-103, 105, 108, 111, 112, 115, 116)	22	339 / 2616	21.0 (13.3-29.8)	94

LC: Long-COVID. CI: Confidence interval. n/N^A: number of subjects with any specific symptom / total number of subjects with Long-COVID.

Table S5. Results of the head-to-head meta-analyses showing the association between each demographic and clinical characteristic and the likelihood of developing Long-COVID. Data from single datasets have been combined using a random-effect model. Overall and stratified analyses were computed after excluding studies that analyzed the RECOVER cohort; see the main text for further details.

Outcomes	**N. datasets (sample)**	**Pooled OR (95% CI)**	***p***	**I^2,%**
(a) Gender, females vs. males, excluding RECOVER cohort (33, 74, 88, 90, 96, 99, 102, 107, 109, 113-116)	16 (112,313)	1.05 (0.98-1.12)	0.2	22
(b) Age-class:				
- 0-5y (33, 51, 73, 90, 96, 109, 111, 113-115)		1 (ref. cat.)	--	--
- 6-11y Excluding RECOVER cohort (33, 73, 90, 96, 109, 111, 113, 115)	8 (271,780)	1.46 (1.13-1.89)	<0.01	20
- 12-18y (33, 73, 90, 96, 109, 111, 113-115)	12 (73,737)	1.75 (1.38-2.23)	<0.001	92
(c) Presence of comorbidities, yes vs. no, excluding RECOVER cohort (33, 74, 88, 90, 92, 93, 96, 102, 107, 109, 112-114)	16 (96,962)	1.74 (1.56-1.94)	<0.001	64
(d) Symptomatic SARS-CoV-2 infection vs. asymptomatic (33, 74, 80, 90, 92, 96, 99, 116)	8 (4892)	2.47 (1.37-4.46)	<0.01	88
(e) Severe COVID-19 [A],yes vs. no, excluding RECOVER cohort (33, 80, 85, 90, 92, 96, 102, 114)	11 (94,597)	1.90 (1.41-2.56)	<0.001	85
(f) SARS-CoV-2 vaccinated individuals [B], vs. unvaccinated, excluding RECOVER cohort (33, 93, 99, 102, 116)	5 (33,187)	0.87 (0.48-1.59)	0.7	55

OR: odds ratio; CI: confidence interval.

[A] Severe COVID-19 is a symptomatic disease requiring hospital admission during primary infection.

[B] At least 1 vaccine dose, vs. none.

Table S6. Quality evaluation of the 52 included studies, according to the Newcastle-Ottawa Quality Assessment Scale for cohort studies[A]

First author	Selection	Comparability	Outcome
Smane 2020 (72)	2	0	1
Blomberg 2021 (78)	4	2	3
Fink 2021 (79)	3	2	2
Matteudi 2021 (80) *	2	1	2
Molteni 2021 (81)	3	1	2
Radtke 2021 (82)	4	1	2
Roge 2021 (12)	3	1	2
Say 2021 (83)	3	0	2
Sterky 2021 (84)	2	0	2
Bergia 2022 (85) *	4	1	2
Bloise 2022 (86)	3	0	1
Borch 2022 (87)	3	2	1
Buonsenso 2022 (15)	4	2	1
Dumont 2022 (88) *	4	2	1
Erol 2022 (89)	2	1	2
Funk 2022 (90) *	4	2	2
Guven 2022 (91)	4	1	3
Maddux 2022 (92) *	3	1	1
Messiah 2022 (93) *	3	1	2
Miller 2022 (94)	3	2	1
Osmanov 2022 (73) *	3	0	1
Pazukhina 2022 (74) *	4	0	1
Roessler 2022 (95)	3	2	3
Trapani 2022 (96) *	4	0	1

(continued)

(continued)

First author	Selection	Comparability	Outcome
Zavala 2022 (97)	4	2	1
de Lima 2023 (98)	3	0	1
Ertesvåg 2023 (99) *	3	0	1
Jarupan 2023 (100)	3	0	2
Körner 2023 (101)	2	0	1
Li 2023 (102) *	3	2	1
Mancino 2023 (103)	3	0	1
Pinto Pereira A 2023 (37)	4	2	1
Pinto Pereira B 2023 (49)	4	2	1
Sedik 2023 (104)	3	0	2
Seery 2023 (105)	4	2	1
Warren-Gash 2023 (106)	4	2	1
Boyarchuk 2024 (107) *	3	0	2
Calcaterra 2024 (108)	3	0	1
Camporesi 2024 (33) *	2	0	2
Kostev 2024 (109) *	2	0	2
Sansone 2024 (110)	2	1	2
Sarani 2024 (111) *	4	2	2
Stephenson 2024 (75)	3	2	1
Wongwathanavikrom 2024 (112) *	3	1	3
Britton 2025 (113) *	2	0	2
Dixon 2025 (114) *	2	2	3
Esposito 2025 (115) *	3		2
Gross 2025 (76)	3	2	2
Iijima 2025 (116) *	4	0	2

(continued)

(continued)

First author	Selection	Comparability	Outcome
Mandel 2025 (51) *	3	1	3
Rao 2025 (77)	2	2	2
Yang 2025 (117)	1	0	1

* 21 studies included also in the head to head meta-analyses.

[A] Studies are ranked as good, fair, or poor quality according to the Newcastle–Ottawa Scale star system, as follows:

Good quality: 3/4 stars in selection domain AND 1/2 stars in comparability domain AND 2/3 stars in outcome domain.

Fair quality: 2 stars in selection domain AND 1/2 stars in comparability domain AND 2/3 stars in outcome domain.

Poor quality: 0/1 stars in selection domain AND 0 stars in comparability domain AND 0/1 stars in outcome domain.

Table S7: PRISMA 2020 Checklist

Section and Topic	Item #	Checklist item	Location where item is reported
**TITLE**			
Title	1	Identify the report as a systematic review.	Lines 2-3
**ABSTRACT**			
Abstract	2	See the PRISMA 2020 for Abstracts checklist.	Lines 14-35
**INTRODUCTION**			
Rationale	3	Describe the rationale for the review in the context of existing knowledge.	Lines 43-62
Objectives	4	Provide an explicit statement of the objective(s) or question(s) the review addresses.	Lines 63-67
**METHODS**			
Eligibility criteria	5	Specify the inclusion and exclusion criteria for the review and how studies were grouped for the syntheses.	Lines 77-102
Information sources	6	Specify all databases, registers, websites, organisations, reference lists and other sources searched or consulted to identify studies. Specify the date when each source was last searched or consulted.	Lines 71-75
Search strategy	7	Present the full search strategies for all databases, registers and websites, including any filters and limits used.	Lines 73-74
Selection process	8	Specify the methods used to decide whether a study met the inclusion criteria of the review, including how many reviewers screened each record and each report retrieved, whether they worked independently, and if applicable, details of automation tools used in the process.	Lines 71-72
Data collection process	9	Specify the methods used to collect data from reports, including how many reviewers collected data from each report, whether they worked independently, any processes for obtaining or confirming data from study investigators, and if applicable, details of automation tools used in the process.	Lines 71-72 and lines 100-102
Data items	10a	List and define all outcomes for which data were sought. Specify whether all results that were compatible with each outcome domain in each study were sought (e.g. for all measures, time points, analyses), and if not, the methods used to decide which results to collect.	Lines 104-141
	10b	List and define all other variables for which data were sought (e.g. participant and intervention characteristics, funding sources). Describe any assumptions made about any missing or unclear information.	Lines 141-146
Study risk of bias assessment	11	Specify the methods used to assess risk of bias in the included studies, including details of the tool(s) used, how many reviewers assessed each study and whether they worked independently, and if applicable, details of automation tools used in the process.	Lines 225-226
Effect measures	12	Specify for each outcome the effect measure(s) (e.g. risk ratio, mean difference) used in the synthesis or presentation of results.	Lines 104-105<br>Lines 146-148
Synthesis methods	13a	Describe the processes used to decide which studies were eligible for each synthesis (e.g. tabulating the study intervention characteristics and comparing against the planned groups for each synthesis (item #5)).	Table 1
	13b	Describe any methods required to prepare the data for presentation or synthesis, such as handling of missing summary statistics, or data	Lines 143-146

Section and Topic	Item #	Checklist item	Location where item is reported
		conversions.	
	13c	Describe any methods used to tabulate or visually display results of individual studies and syntheses.	Lines 143-146; supplementary figures S1-S7
	13d	Describe any methods used to synthesize results and provide a rationale for the choice(s). If meta-analysis was performed, describe the model(s), method(s) to identify the presence and extent of statistical heterogeneity, and software package(s) used.	Lines 104-141 Lines 146-153
	13e	Describe any methods used to explore possible causes of heterogeneity among study results (e.g. subgroup analysis, meta-regression).	Lines 150-151
	13f	Describe any sensitivity analyses conducted to assess robustness of the synthesized results.	Lines 195-201
Reporting bias assessment	14	Describe any methods used to assess risk of bias due to missing results in a synthesis (arising from reporting biases).	Lines 225-226
Certainty assessment	15	Describe any methods used to assess certainty (or confidence) in the body of evidence for an outcome.	Lines 147-148
**RESULTS**			
Study selection	16a	Describe the results of the search and selection process, from the number of records identified in the search to the number of studies included in the review, ideally using a flow diagram.	Lines 156-159; figure 1
	16b	Cite studies that might appear to meet the inclusion criteria, but which were excluded, and explain why they were excluded.	Lines 160-161; supplementary table S1
Study characteristics	17	Cite each included study and present its characteristics.	Table 1
Risk of bias in studies	18	Present assessments of risk of bias for each included study.	Supplementary table S2
Results of individual studies	19	For all outcomes, present, for each study: (a) summary statistics for each group (where appropriate) and (b) an effect estimate and its precision (e.g. confidence/credible interval), ideally using structured tables or plots.	Tables 2-3-4
Results of syntheses	20a	For each synthesis, briefly summarise the characteristics and risk of bias among contributing studies.	Supplementary table S2
	20b	Present results of all statistical syntheses conducted. If meta-analysis was done, present for each the summary estimate and its precision (e.g. confidence/credible interval) and measures of statistical heterogeneity. If comparing groups, describe the direction of the effect.	Lines 181-223
	20c	Present results of all investigations of possible causes of heterogeneity among study results.	Tables 2-3-4

Section and Topic	Item #	Checklist item	Location where item is reported
	20d	Present results of all sensitivity analyses conducted to assess the robustness of the synthesized results.	Supplementary tables S1-S2- S3
Reporting biases	21	Present assessments of risk of bias due to missing results (arising from reporting biases) for each synthesis assessed.	Lines 234-237; supplementary figure S8
Certainty of evidence	22	Present assessments of certainty (or confidence) in the body of evidence for each outcome assessed.	Tables 2-3-4
**DISCUSSION**			
Discussion	23a	Provide a general interpretation of the results in the context of other evidence.	Lines 298-322
	23b	Discuss any limitations of the evidence included in the review.	Lines 349-358
	23c	Discuss any limitations of the review processes used.	Lines 339-348
	23d	Discuss implications of the results for practice, policy, and future research.	Lines 359-365
**OTHER INFORMATION**			
Registration and protocol	24a	Provide registration information for the review, including register name and registration number, or state that the review was not registered.	Not registered
	24b	Indicate where the review protocol can be accessed, or state that a protocol was not prepared.	Not prepared
	24c	Describe and explain any amendments to information provided at registration or in the protocol.	Not applicable
Support	25	Describe sources of financial or non-financial support for the review, and the role of the funders or sponsors in the review.	Line 395
Competing interests	26	Declare any competing interests of review authors.	Line 400
Availability of data, code and other materials	27	Report which of the following are publicly available and where they can be found: template data collection forms; data extracted from included studies; data used for all analyses; analytic code; any other materials used in the review.	Lines 398-399

Figure S1. Likelihood of developing Long-COVID in female versus male subjects with a previous history of laboratory-confirmed SARS-CoV-2 infection. Data from single studies have been combined adopting a random-effect model.

Study or Subgroup	log[Odds Ratio]	SE	Weight	Odds Ratio IV, Random, 95% CI	Year
Trapani 2022	0.1655	0.2047	2.7%	1.18 [0.79, 1.76]	2022
Dumont 2022	-0.0943	0.2477	1.9%	0.91 [0.56, 1.48]	2022
Funk 2022	0.3221	0.2069	2.7%	1.38 [0.92, 2.07]	2022
Pazukhina 2022	0.157	0.358	1.0%	1.17 [0.58, 2.36]	2022
Li 2023	-0.5276	0.2664	1.7%	0.59 [0.35, 0.99]	2023
Ertesvåg 2023	0.1989	0.3135	1.2%	1.22 [0.66, 2.26]	2023
Camporesi 2024	0.077	0.267	1.7%	1.08 [0.64, 1.82]	2024
Boyarchuk 2024	0.7275	0.3319	1.1%	2.07 [1.08, 3.97]	2024
Kostev 2024	0.1655	0.1983	2.9%	1.18 [0.80, 1.74]	2024
Esposito 2025	0.0488	0.1517	4.5%	1.05 [0.78, 1.41]	2025
Iijima 2025	0.3577	0.4969	0.5%	1.43 [0.54, 3.79]	2025
Mandel 2025	0.1655	0.0176	20.9%	1.18 [1.14, 1.22]	2025
Dixon Arizona 2025	0	0.0481	15.8%	1.00 [0.91, 1.10]	2025
Dixon Bronx 2025	-0.0202	0.0549	14.6%	0.98 [0.88, 1.09]	2025
Dixon Indinana 2025	0.077	0.0242	20.0%	1.08 [1.03, 1.13]	2025
Dixon Utah 2025	-0.1165	0.1449	4.8%	0.89 [0.67, 1.18]	2025
Britton 2025	0.3716	0.2433	2.0%	1.45 [0.90, 2.34]	2025
**Total (95% CI)**			**100.0%**	**1.08 [1.00, 1.16]**	

Heterogeneity: Tau² = 0.01; Chi² = 37.74, df = 16 (P = 0.002); I² = 58%
Test for overall effect: Z = 2.03 (P = 0.04)

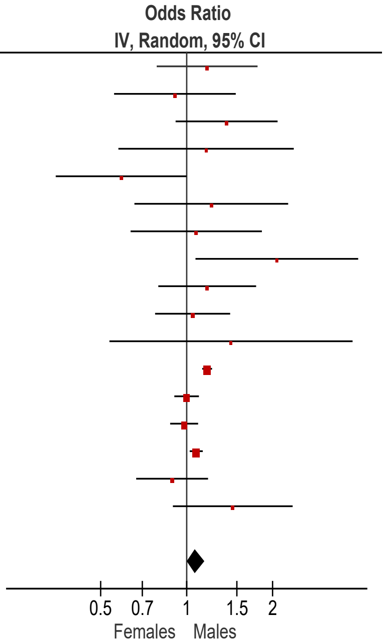

Sensitivity analyses:
High quality studies only

Study or Subgroup	log[Odds Ratio]	SE	Weight	Odds Ratio IV, Random, 95% CI	Year
Funk 2022	0.3221	0.2069	29.5%	1.38 [0.92, 2.07]	2022
Li 2023	-0.5276	0.2664	23.7%	0.59 [0.35, 0.99]	2023
Mandel 2025	0.1655	0.0176	46.9%	1.18 [1.14, 1.22]	2025
**Total (95% CI)**			**100.0%**	**1.05 [0.73, 1.50]**	

Heterogeneity: Tau² = 0.07; Chi² = 7.33, df = 2 (P = 0.03); I² = 73%
Test for overall effect: Z = 0.26 (P = 0.80)

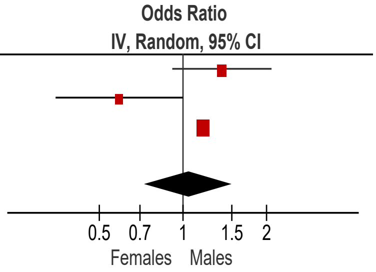

Excluding RECOVER cohort

Study or Subgroup	log[Odds Ratio]	SE	Weight	Odds Ratio IV, Random, 95% CI	Year
Dumont 2022	-0.0943	0.2477	1.7%	0.91 [0.56, 1.48]	2022
Funk 2022	0.3221	0.2069	2.4%	1.38 [0.92, 2.07]	2022
Pazukhina 2022	0.157	0.358	0.8%	1.17 [0.58, 2.36]	2022
Trapani 2022	0.1655	0.2047	2.5%	1.18 [0.79, 1.76]	2022
Li 2023	-0.5276	0.2664	1.5%	0.59 [0.35, 0.99]	2023
Ertesvåg 2023	0.1989	0.3135	1.1%	1.22 [0.66, 2.26]	2023
Camporesi 2024	0.077	0.267	1.5%	1.08 [0.64, 1.82]	2024
Boyarchuk 2024	0.7275	0.3319	1.0%	2.07 [1.08, 3.97]	2024
Kostev 2024	0.1655	0.1983	2.6%	1.18 [0.80, 1.74]	2024
Dixon Arizona 2025	0	0.0481	21.7%	1.00 [0.91, 1.10]	2025
Dixon Bronx 2025	-0.0202	0.0549	19.1%	0.98 [0.88, 1.09]	2025
Dixon Indinana 2025	0.077	0.0242	32.9%	1.08 [1.03, 1.13]	2025
Dixon Utah 2025	-0.1165	0.1449	4.6%	0.89 [0.67, 1.18]	2025
Britton 2025	0.3716	0.2433	1.8%	1.45 [0.90, 2.34]	2025
Esposito 2025	0.0488	0.1517	4.3%	1.05 [0.78, 1.41]	2025
Iijima 2025	0.3577	0.4969	0.4%	1.43 [0.54, 3.79]	2025
**Total (95% CI)**			**100.0%**	**1.05 [0.98, 1.12]**	

Heterogeneity: Tau² = 0.00; Chi² = 19.29, df = 15 (P = 0.20); I² = 22%
Test for overall effect: Z = 1.35 (P = 0.18)

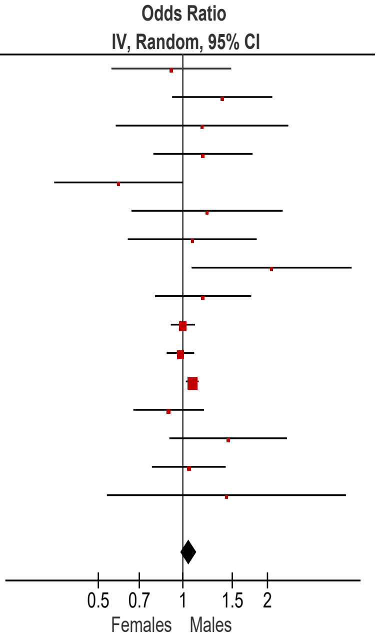

Figure S2. Likelihood of developing Long-COVID in children (6 to 11 years old) versus infants (0 to 5 years old) with a previous history of laboratory-confirmed SARS-CoV-2 infection. Data from single datasets have been combined adopting a random-effect model.

Study or Subgroup	log[Odds Ratio]	SE	Weight	Odds Ratio IV, Random, 95% CI	Year
Trapani 2022	0.3436	0.2643	12.4%	1.41 [0.84, 2.37]	2022
Funk 2022	0.3365	0.3464	9.2%	1.40 [0.71, 2.76]	2022
Osmanov 2022	0.9439	0.3517	9.0%	2.57 [1.29, 5.12]	2022
Kostev 2024	0.4511	0.2617	12.5%	1.57 [0.94, 2.62]	2024
Camporesi 2024	0.0953	0.4231	7.0%	1.10 [0.48, 2.52]	2024
Sarani 2024	1.1569	0.5752	4.4%	3.18 [1.03, 9.82]	2024
Britton 2025	0.5068	0.3724	8.4%	1.66 [0.80, 3.44]	2025
Mandel 2025	-0.0101	0.0264	24.0%	0.99 [0.94, 1.04]	2025
Esposito 2025	-0.1054	0.2513	13.0%	0.90 [0.55, 1.47]	2025
**Total (95% CI)**			**100.0%**	**1.35 [1.04, 1.75]**	

Heterogeneity: Tau² = 0.07; Chi² = 18.96, df = 8 (P = 0.02); I² = 58%
Test for overall effect: Z = 2.24 (P = 0.02)

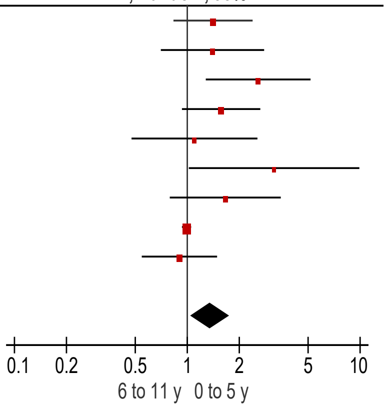

Sensitivity analyses:
High quality studies only

Study or Subgroup	log[Odds Ratio]	SE	Weight	Odds Ratio IV, Random, 95% CI	Year
Funk 2022	0.3365	0.3464	29.3%	1.40 [0.71, 2.76]	2022
Sarani 2024	1.1569	0.5752	16.1%	3.18 [1.03, 9.82]	2024
Mandel 2025	-0.0101	0.0264	54.7%	0.99 [0.94, 1.04]	2025
**Total (95% CI)**			**100.0%**	**1.32 [0.77, 2.26]**	

Heterogeneity: Tau² = 0.14; Chi² = 5.09, df = 2 (P = 0.08); I² = 61%
Test for overall effect: Z = 1.02 (P = 0.31)

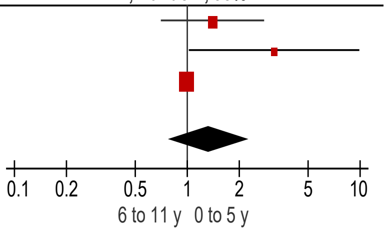

Excluding RECOVER color

Study or Subgroup	log[Odds Ratio]	SE	Weight	Odds Ratio IV, Random, 95% CI	Year
Trapani 2022	0.3436	0.2643	17.5%	1.41 [0.84, 2.37]	2022
Funk 2022	0.3365	0.3464	11.5%	1.40 [0.71, 2.76]	2022
Osmanov 2022	0.9439	0.3517	11.3%	2.57 [1.29, 5.12]	2022
Kostev 2024	0.4511	0.2617	17.7%	1.57 [0.94, 2.62]	2024
Camporesi 2024	0.0953	0.4231	8.2%	1.10 [0.48, 2.52]	2024
Sarani 2024	1.1569	0.5752	4.7%	3.18 [1.03, 9.82]	2024
Britton 2025	0.5068	0.3724	10.2%	1.66 [0.80, 3.44]	2025
Esposito 2025	-0.1054	0.2513	18.8%	0.90 [0.55, 1.47]	2025
**Total (95% CI)**			**100.0%**	**1.46 [1.13, 1.89]**	

Heterogeneity: Tau² = 0.03; Chi² = 8.78, df = 7 (P = 0.27); I² = 20%
Test for overall effect: Z = 2.92 (P = 0.004)

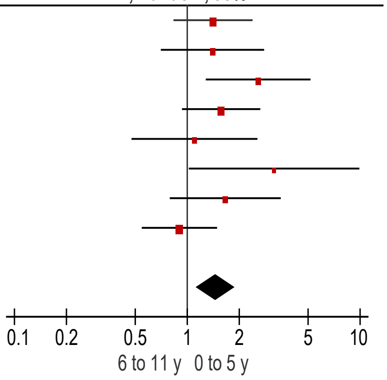

Figure S3. Likelihood of developing Long-COVID in adolescents (12 years old) versus infants (< 5 years old) with a previous history of laboratory-confirmed SARS-CoV-2 infection. Data from single datasets have been combined adopting a random-effect model.

Study or Subgroup	log[Odds Ratio]	SE	Weight	Odds Ratio IV, Random, 95% CI	Year
Funk 2022	0.8286	0.2332	8.7%	2.29 [1.45, 3.62]	2022
Osmanov 2022	0.9243	0.3222	6.8%	2.52 [1.34, 4.74]	2022
Trapani 2022	0.7793	0.2599	8.1%	2.18 [1.31, 3.63]	2022
Kostev 2024	1.1442	0.3101	7.0%	3.14 [1.71, 5.77]	2024
Camporesi 2024	1.3083	0.4341	4.9%	3.70 [1.58, 8.66]	2024
Sarani 2024	1.5195	0.6036	3.1%	4.57 [1.40, 14.92]	2024
Dixon Utah 2025	0.4318	0.1625	10.3%	1.54 [1.12, 2.12]	2025
Esposito 2025	0.1823	0.2823	7.6%	1.20 [0.69, 2.09]	2025
Dixon Arizona 2025	-0.2107	0.0601	12.3%	0.81 [0.72, 0.91]	2025
Dixon Bronx 2025	-0.0101	0.0601	12.3%	0.99 [0.88, 1.11]	2025
Dixon Indinana 2025	0.3293	0.0302	12.5%	1.39 [1.31, 1.47]	2025
Britton 2025	1.5129	0.3382	6.5%	4.54 [2.34, 8.81]	2025
**Total (95% CI)**			**100.0%**	**1.75 [1.38, 2.23]**	

Heterogeneity: Tau² = 0.12; Chi² = 131.47, df = 11 (P < 0.00001); I² = 92%

Test for overall effect: Z = 4.55 (P < 0.00001)

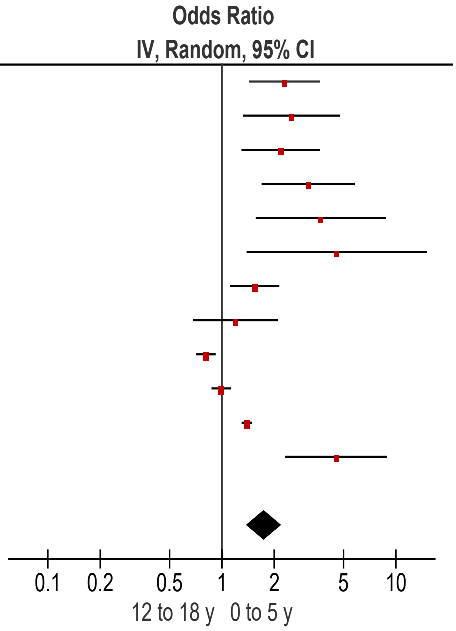

Figure S4. Likelihood of developing Long-COVID among SARS-CoV-2 positive subjects with at least one comorbidity, versus subjects without a history of comorbidities. Data from single datasets have been combined adopting a random-effect model.

Study or Subgroup	log[Odds Ratio]	SE	Weight	Odds Ratio IV, Random, 95% CI	Year
Pazukhina 2022	1.1506	0.3163	4.2%	3.16 [1.70, 5.87]	2022
Trapani 2022	0.1044	0.3312	4.0%	1.11 [0.58, 2.12]	2022
Maddux 2022	0.7975	0.5082	2.2%	2.22 [0.82, 6.01]	2022
Messiah 2022	0.4947	0.2732	5.0%	1.64 [0.96, 2.80]	2022
Dumont 2022	1.2528	0.2855	4.8%	3.50 [2.00, 6.12]	2022
Funk 2022	0.0392	0.2639	5.2%	1.04 [0.62, 1.74]	2022
Li 2023	-0.5447	0.4294	2.8%	0.58 [0.25, 1.35]	2023
Camporesi 2024	0.1398	0.3671	3.5%	1.15 [0.56, 2.36]	2024
Wongwathanavikrom 2024	1.3987	0.5568	1.9%	4.05 [1.36, 12.06]	2024
Kostev 2024	0.3365	0.1468	7.9%	1.40 [1.05, 1.87]	2024
Boyarchuk 2024	0.9083	0.3415	3.9%	2.48 [1.27, 4.84]	2024
Britton 2025	0.7419	0.2447	5.6%	2.10 [1.30, 3.39]	2025
Mandel 2025	0.1823	0.0173	10.4%	1.20 [1.16, 1.24]	2025
Dixon Arizona 2025	0.6419	0.0449	10.1%	1.90 [1.74, 2.07]	2025
Dixon Bronx 2025	0.4383	0.0483	10.1%	1.55 [1.41, 1.70]	2025
Dixon Indinana 2025	0.5988	0.0229	10.3%	1.82 [1.74, 1.90]	2025
Dixon Utah 2025	0.6206	0.1377	8.2%	1.86 [1.42, 2.44]	2025
**Total (95% CI)**			**100.0%**	**1.68 [1.42, 1.98]**	

Heterogeneity: Tau² = 0.07; Chi² = 291.49, df = 16 (P < 0.00001); I² = 95%
Test for overall effect: Z = 6.09 (P < 0.00001)

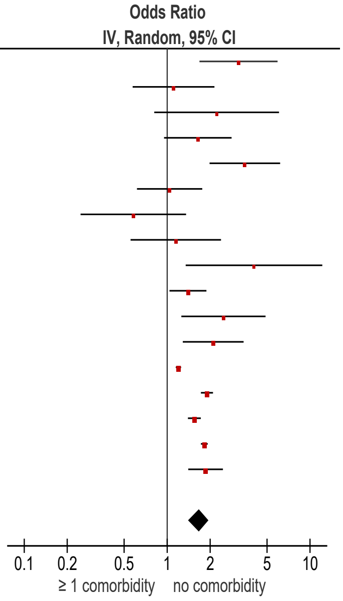

Sensitivity analyses:
High quality studies only

Study or Subgroup	log[Odds Ratio]	SE	Weight	Odds Ratio IV, Random, 95% CI	Year
Messiah 2022	0.4947	0.2732	19.9%	1.64 [0.96, 2.80]	2022
Funk 2022	0.0392	0.2639	20.6%	1.04 [0.62, 1.74]	2022
Li 2023	-0.5447	0.4294	11.4%	0.58 [0.25, 1.35]	2023
Wongwathanavikrom 2024	1.3987	0.5568	7.7%	4.05 [1.36, 12.06]	2024
Mandel 2025	0.1823	0.0173	40.3%	1.20 [1.16, 1.24]	2025
**Total (95% CI)**			**100.0%**	**1.25 [0.90, 1.75]**	

Heterogeneity: Tau² = 0.07; Chi² = 9.24, df = 4 (P = 0.06); I² = 57%
Test for overall effect: Z = 1.32 (P = 0.19)

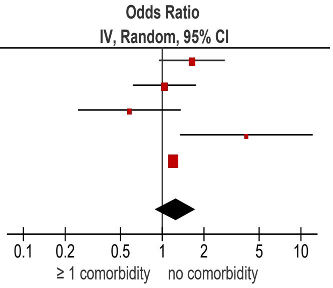

Excluding RECOVER color

Study or Subgroup	log[Odds Ratio]	SE	Weight	Odds Ratio IV, Random, 95% CI	Year
Pazukhina 2022	1.1506	0.3163	2.7%	3.16 [1.70, 5.87]	2022
Trapani 2022	0.1044	0.3312	2.5%	1.11 [0.58, 2.12]	2022
Maddux 2022	0.7975	0.5082	1.1%	2.22 [0.82, 6.01]	2022
Messiah 2022	0.4947	0.2732	3.5%	1.64 [0.96, 2.80]	2022
Dumont 2022	1.2528	0.2855	3.2%	3.50 [2.00, 6.12]	2022
Funk 2022	0.0392	0.2639	3.7%	1.04 [0.62, 1.74]	2022
Li 2023	-0.5447	0.4294	1.6%	0.58 [0.25, 1.35]	2023
Camporesi 2024	0.1398	0.3671	2.1%	1.15 [0.56, 2.36]	2024
Wongwathanavikrom 2024	1.3987	0.5568	1.0%	4.05 [1.36, 12.06]	2024
Kostev 2024	0.3365	0.1468	8.4%	1.40 [1.05, 1.87]	2024
Boyarchuk 2024	0.9083	0.3415	2.4%	2.48 [1.27, 4.84]	2024
Dixon Arizona 2025	0.6419	0.0449	17.8%	1.90 [1.74, 2.07]	2025
Dixon Bronx 2025	0.4383	0.0483	17.5%	1.55 [1.41, 1.70]	2025
Dixon Indinana 2025	0.5988	0.0229	19.5%	1.82 [1.74, 1.90]	2025
Dixon Utah 2025	0.6206	0.1377	9.0%	1.86 [1.42, 2.44]	2025
Britton 2025	0.7419	0.2447	4.1%	2.10 [1.30, 3.39]	2025
**Total (95% CI)**			**100.0%**	**1.74 [1.56, 1.94]**	

Heterogeneity: Tau² = 0.02; Chi² = 41.19, df = 15 (P = 0.0003); I² = 64%
Test for overall effect: Z = 9.92 (P < 0.00001)

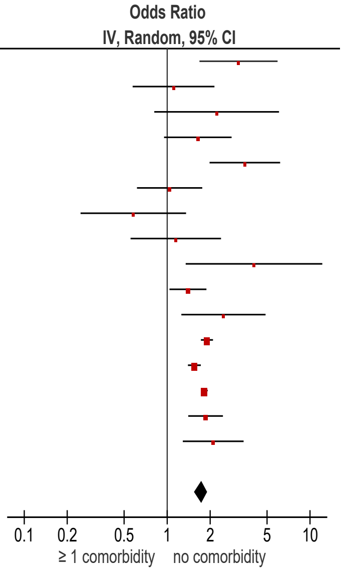

Figure S5. Likelihood of developing Long-COVID among subjects with a previous history of symptomatic COVID-19 versus subjects with a previous asymptomatic SARS-CoV-2 infection. Data from single datasets have been combined adopting a random-effect model.

Study or Subgroup	log[Odds Ratio]	SE	Weight	Odds Ratio IV, Random, 95% CI	Year
Matteudi 2021	1.5686	0.7611	8.3%	4.80 [1.08, 21.33]	2021
Funk 2022	1.1878	0.2179	16.0%	3.28 [2.14, 5.03]	2022
Maddux 2022	0.2546	0.1099	17.1%	1.29 [1.04, 1.60]	2022
Pazukhina 2022	0.9933	0.903	6.8%	2.70 [0.46, 15.85]	2022
Trapani 2022	1.8825	0.2092	16.2%	6.57 [4.36, 9.90]	2022
Ertesvåg 2023	0.2776	0.7559	8.4%	1.32 [0.30, 5.81]	2023
Camporesi 2024	0.8629	0.265	15.4%	2.37 [1.41, 3.98]	2024
Iijima 2025	0.0953	0.5035	11.8%	1.10 [0.41, 2.95]	2025
**Total (95% CI)**			**100.0%**	**2.47 [1.37, 4.46]**	

Heterogeneity: $Tau^2 = 0.52$; $Chi^2 = 56.91$, $df = 7$ ($P < 0.00001$); $I^2 = 88\%$
Test for overall effect: $Z = 2.99$ ($P = 0.003$)

Odds Ratio
IV, Random, 95% CI

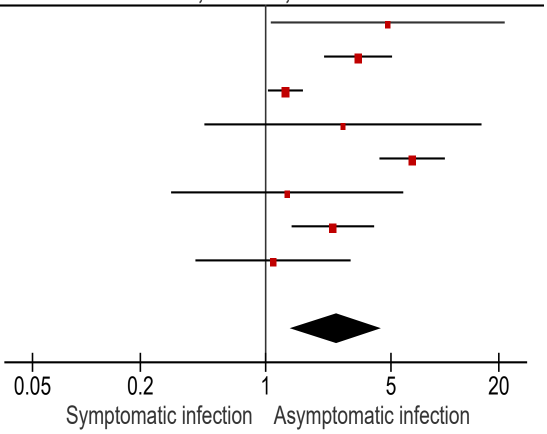

Figure S6. Likelihood of developing Long-COVID among subjects with a severe COVID-19 requiring hospitalization versus subjects not requiring hospital admission during the primary infection. Data from single datasets have been combined adopting a random-effect model.

Study or Subgroup	log[Odds Ratio]	SE	Weight	Odds Ratio IV, Random, 95% CI	Year
Matteudi 2021	1.0006	0.6642	1.9%	2.72 [0.74, 10.00]	2021
Funk 2022	0.9042	0.2089	9.5%	2.47 [1.64, 3.72]	2022
Maddux 2022	-0.5447	0.4294	3.9%	0.58 [0.25, 1.35]	2022
Trapani 2022	0.9123	0.3086	6.2%	2.49 [1.36, 4.56]	2022
Bergia 2022	1.3913	0.2444	8.2%	4.02 [2.49, 6.49]	2022
Li 2023	0.5365	0.5976	2.3%	1.71 [0.53, 5.52]	2023
Camporesi 2024	1.8764	0.7471	1.5%	6.53 [1.51, 28.24]	2024
Dixon Arizona 2025	-0.1985	0.1345	12.8%	0.82 [0.63, 1.07]	2025
Dixon Bronx 2025	0.5365	0.0912	14.7%	1.71 [1.43, 2.04]	2025
Dixon Indinana 2025	0.7467	0.0562	16.0%	2.11 [1.89, 2.36]	2025
Dixon Utah 2025	0.7178	0.313	6.1%	2.05 [1.11, 3.79]	2025
Mandel 2025	0.6981	0.0155	16.9%	2.01 [1.95, 2.07]	2025
**Total (95% CI)**			**100.0%**	**1.88 [1.55, 2.27]**	

Heterogeneity: Tau² = 0.06; Chi² = 68.65, df = 11 (P < 0.00001); I² = 84%
Test for overall effect: Z = 6.49 (P < 0.00001)

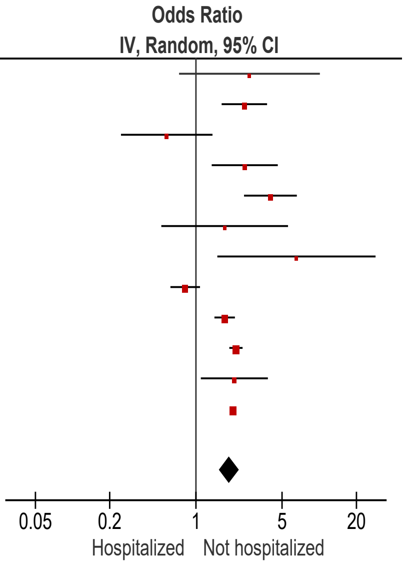

Sensitivity analyses:
High quality studies only

Study or Subgroup	log[Odds Ratio]	SE	Weight	Odds Ratio IV, Random, 95% CI	Year
Funk 2022	0.9042	0.2089	0.5%	2.47 [1.64, 3.72]	2022
Li 2023	0.5365	0.5976	0.1%	1.71 [0.53, 5.52]	2023
Mandel 2025	0.6981	0.0155	99.4%	2.01 [1.95, 2.07]	2025
**Total (95% CI)**			**100.0%**	**2.01 [1.95, 2.07]**	

Heterogeneity: Tau² = 0.00; Chi² = 1.04, df = 2 (P = 0.59); I² = 0%
Test for overall effect: Z = 45.24 (P < 0.00001)

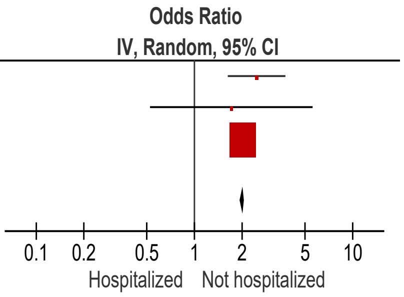

Excluding RECOVER cohort

Study or Subgroup	log[Odds Ratio]	SE	Weight	Odds Ratio IV, Random, 95% CI	Year
Matteudi 2021	1.0006	0.6642	3.9%	2.72 [0.74, 10.00]	2021
Trapani 2022	0.9123	0.3086	9.1%	2.49 [1.36, 4.56]	2022
Bergia 2022	1.3913	0.2444	10.6%	4.02 [2.49, 6.49]	2022
Funk 2022	0.9042	0.2089	11.5%	2.47 [1.64, 3.72]	2022
Maddux 2022	-0.5447	0.4294	6.8%	0.58 [0.25, 1.35]	2022
Li 2023	0.5365	0.5976	4.5%	1.71 [0.53, 5.52]	2023
Camporesi 2024	1.8764	0.7471	3.2%	6.53 [1.51, 28.24]	2024
Dixon Indinana 2025	0.7467	0.0562	14.3%	2.11 [1.89, 2.36]	2025
Dixon Utah 2025	0.7178	0.313	9.0%	2.05 [1.11, 3.79]	2025
Dixon Arizona 2025	-0.1985	0.1345	13.1%	0.82 [0.63, 1.07]	2025
Dixon Bronx 2025	0.5365	0.0912	13.9%	1.71 [1.43, 2.04]	2025
**Total (95% CI)**			**100.0%**	**1.90 [1.41, 2.56]**	

Heterogeneity: Tau² = 0.16; Chi² = 66.25, df = 10 (P < 0.00001); I² = 85%
Test for overall effect: Z = 4.21 (P < 0.0001)

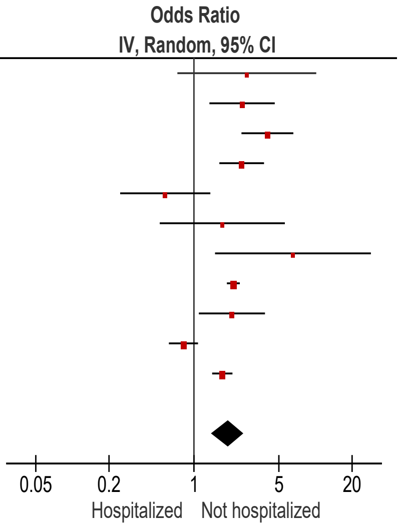

Figure S7. Likelihood of developing Long-COVID among subjects receiving ≥1 anti-SARS-CoV-2 vaccine dose, versus unvaccinated individuals. Data from single datasets have been combined adopting a random-effect model.

Study or Subgroup	log[Odds Ratio]	SE	Weight	Odds Ratio IV, Random, 95% CI	Year
Messiah 2022	-0.8916	0.3414	18.2%	0.41 [0.21, 0.80]	2022
Li 2023	-0.4308	0.3306	18.8%	0.65 [0.34, 1.24]	2023
Ertesvåg 2023	0.6831	0.5305	11.0%	1.98 [0.70, 5.60]	2023
Camporesi 2024	0.3001	0.431	14.3%	1.35 [0.58, 3.14]	2024
Iijima 2025	0.3784	1.068	3.6%	1.46 [0.18, 11.84]	2025
Mandel 2025	0.0953	0.0047	34.0%	1.10 [1.09, 1.11]	2025
**Total (95% CI)**			**100.0%**	**0.92 [0.61, 1.41]**	

Heterogeneity: Tau² = 0.13; Chi² = 12.41, df = 5 (P = 0.03); I² = 60%
Test for overall effect: Z = 0.37 (P = 0.71)

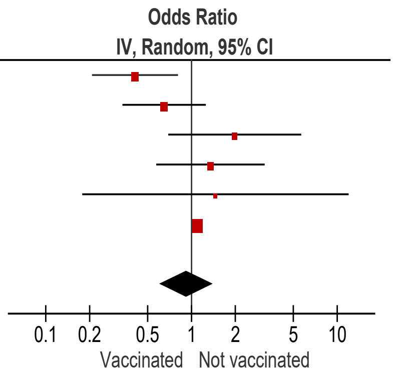

Sensitivity analyses:
High quality studies only

Study or Subgroup	log[Odds Ratio]	SE	Weight	Odds Ratio IV, Random, 95% CI	Year
Messiah 2022	-0.8916	0.3414	28.7%	0.41 [0.21, 0.80]	2022
Li 2023	-0.4308	0.3306	29.3%	0.65 [0.34, 1.24]	2023
Mandel 2025	0.0953	0.0047	42.0%	1.10 [1.09, 1.11]	2025
**Total (95% CI)**			**100.0%**	**0.71 [0.38, 1.34]**	

Heterogeneity: Tau² = 0.25; Chi² = 10.88, df = 2 (P = 0.004); I² = 82%
Test for overall effect: Z = 1.05 (P = 0.29)

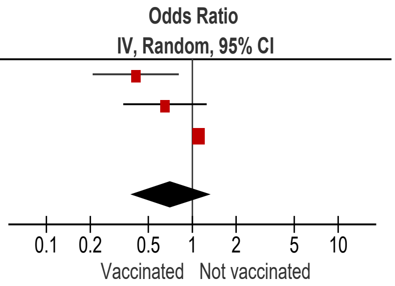

Excluding RECOVER color

Study or Subgroup	log[Odds Ratio]	SE	Weight	Odds Ratio IV, Random, 95% CI	Year
Messiah 2022	-0.8916	0.3414	26.3%	0.41 [0.21, 0.80]	2022
Li 2023	-0.4308	0.3306	26.8%	0.65 [0.34, 1.24]	2023
Ertesvåg 2023	0.6831	0.5305	18.0%	1.98 [0.70, 5.60]	2023
Camporesi 2024	0.3001	0.431	22.0%	1.35 [0.58, 3.14]	2024
Iijima 2025	0.3784	1.068	6.8%	1.46 [0.18, 11.84]	2025
**Total (95% CI)**			**100.0%**	**0.87 [0.48, 1.59]**	

Heterogeneity: Tau² = 0.24; Chi² = 8.90, df = 4 (P = 0.06); I² = 55%
Test for overall effect: Z = 0.44 (P = 0.66)

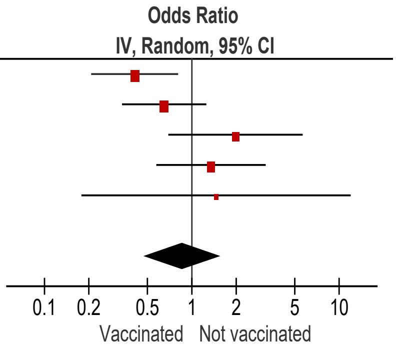

Figure S8. Publication bias assessment; funnel plots and Egger's test for head-to head meta-analyses including ≥10 individual studies. Each point represents an independent study. Evaluated outcomes are risk of developing Long-COVID among: (a) female versus male subjects with a previous history of laboratory-confirmed SARS-CoV-2 infection; (b) adolescents (>12 years old) versus infants (< 5 years old) with a previous history of laboratory-confirmed SARS-CoV-2 infection; (c) SARS-CoV-2 positive subjects with at least one comorbidity, versus subjects without a history of comorbidities; and (d) SARS-CoV-2 positive subjects with a severe COVID-19 requiring hospitalization versus subjects not requiring hospital admission during the primary infection.

(a) Funnel plot of comparison:

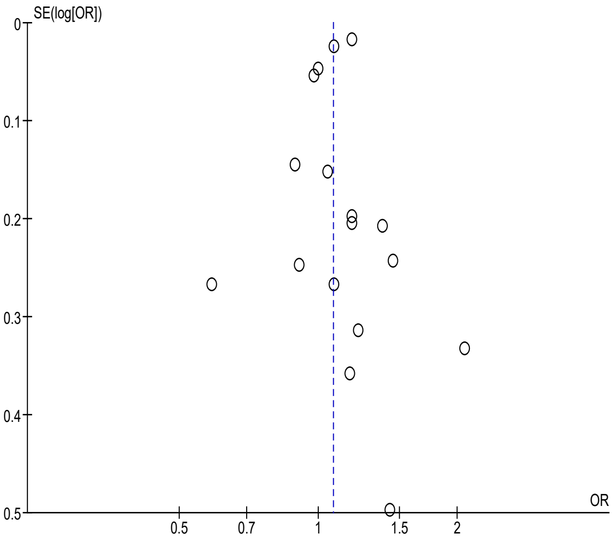

Egger: bias = -0,98 (95% CI: -2,54-0,58) P = 0,20

(b) Funnel plot of comparison:

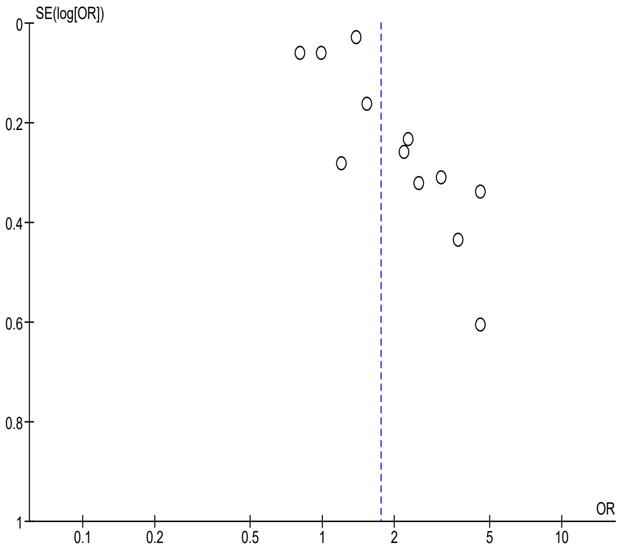

Egger: bias = -0,03 (95% CI: -3,84-3,78) P = 0,98

(c) Funnel plot of comparison:

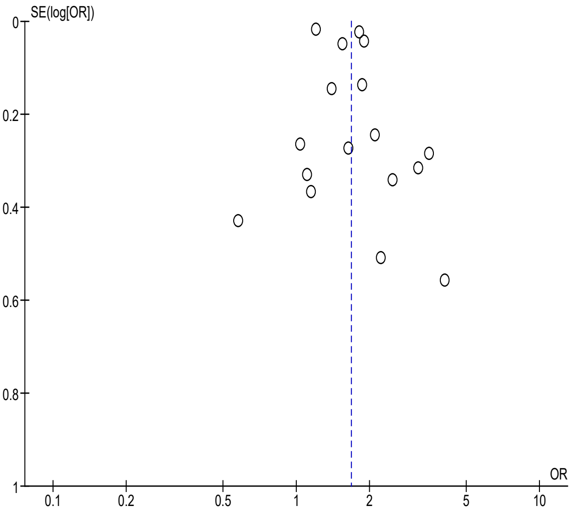

Egger: bias = -0,38 (95% CI: -6,41-5,64) P = 0,89

(d) Funnel plot of comparison:

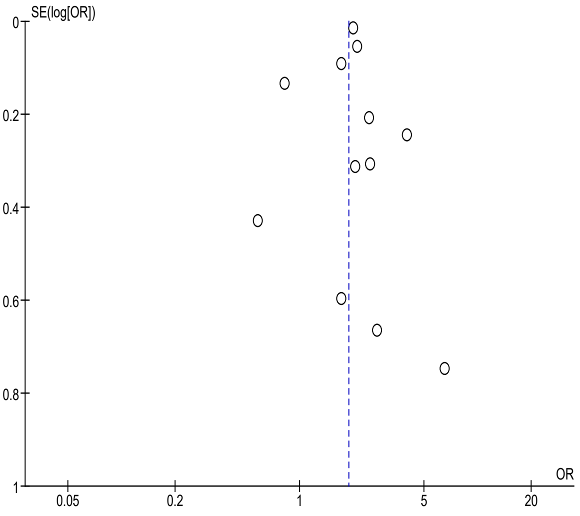

Egger: bias = -0,34 (95% CI: -2,28-1,61) P = 0,70

References

1. Denina M, Pruccoli G, Scolfaro C, Mignone F, Zoppo M, Giraudo I, et al. Sequelae of COVID-19 in Hospitalized Children: A 4-Months Follow-Up. Pediatr Infect Dis J. 2020 Dec;39(12):e458-e9.
2. Asadi-Pooya AA, Nemati H, Shahisavandi M, Akbari A, Emami A, Lotfi M, et al. Long COVID in children and adolescents. World Journal of Pediatrics. 2021 2021/10/01;17(5):495-9.
3. Ashkenazi-Hoffnung L, Shmueli E, Ehrlich S, Ziv A, Bar-On O, Birk E, et al. Long COVID in Children: Observations From a Designated Pediatric Clinic. Pediatr Infect Dis J. 2021 Dec 1;40(12):e509-e11.
4. Brackel CLH, Lap CR, Buddingh EP, van Houten MA, van der Sande L, Langereis EJ, et al. Pediatric long-COVID: An overlooked phenomenon? Pediatr Pulmonol. 2021 Aug;56(8):2495-502.
5. Buonsenso D, Munblit D, De Rose C, Sinatti D, Ricchiuto A, Carfi A, et al. Preliminary evidence on long COVID in children. Acta Paediatr. 2021 Jul;110(7):2208-11.
6. Sante GD, Buonsenso D, De Rose C, Valentini P, Ria F, Sanguinetti M, et al. Immune profile of children with post-acute sequelae of SARS-CoV-2 infection (Long Covid). medRxiv. 2021:2021.05.07.21256539.
7. Leftin Dobkin SC, Collaco JM, McGrath-Morrow SA. Protracted respiratory findings in children post-SARS-CoV-2 infection. Pediatr Pulmonol. 2021 Dec;56(12):3682-7.
8. Ludvigsson JF. Case report and systematic review suggest that children may experience similar long-term effects to adults after clinical COVID-19. Acta Paediatr. 2021 Mar;110(3):914-21.
9. Nogueira Lopez J, Grasa C, Calvo C, Garcia Lopez-Hortelano M. Long-term symptoms of COVID-19 in children. Acta Paediatr. 2021 Jul;110(7):2282-3.
10. Rusetsky Y, Meytel I, Mokoyan Z, Fisenko A, Babayan A, Malyavina U. Smell Status in Children Infected with SARS-CoV-2. Laryngoscope. 2021 Aug;131(8):E2475-E80.
11. Smane L, Roge I, Pucuka Z, Pavare J. Clinical features of pediatric post-acute COVID-19: a descriptive retrospective follow-up study. Ital J Pediatr. 2021 Aug 26;47(1):177.
12. Roge I, Smane L, Kivite-Urtane A, Pucuka Z, Racko I, Klavina L, et al. Comparison of Persistent Symptoms After COVID-19 and Other Non-SARS-CoV-2 Infections in Children. Front Pediatr. 2021;9:752385.
13. Bode SFN, Haendly M, Fabricius D, Mayer B, Zernickel M, Haddad ADM, et al. Pulmonary Function and Persistent Clinical Symptoms in Children and Their Parents 12 Months After Mild SARS-CoV-2 Infection. Front Pediatr. 2022;10:894331.
14. Buonsenso D, Munblit D, Pazukhina E, Ricchiuto A, Sinatti D, Zona M, et al. Post-COVID Condition in Adults and Children Living in the Same Household in Italy: A Prospective Cohort Study Using the ISARIC Global Follow-Up Protocol. Front Pediatr. 2022;10:834875.
15. Buonsenso D, Pazukhina E, Gentili C, Vetrugno L, Morello R, Zona M, et al. The Prevalence, Characteristics and Risk Factors of Persistent Symptoms in Non-Hospitalized and Hospitalized Children with SARS-CoV-2 Infection Followed-Up for up to 12 Months: A Prospective, Cohort Study in Rome, Italy. J Clin Med. 2022 Nov 16;11(22).
16. Buonsenso D, Espuny Pujol F, Munblit D, Pata D, McFarland S, Simpson FK. Clinical characteristics, activity levels and mental health problems in children with long coronavirus disease: a survey of 510 children. Future Microbiol. 2022 May;17(8):577-88.
17. Di Gennaro L, Valentini P, Sorrentino S, Ferretti MA, De Candia E, Basso M, et al. Extended coagulation profile of children with Long Covid: a prospective study. Sci Rep. 2022 Nov 1;12(1):18392.

18. Dolezalova K, Tukova J, Pohunek P. The respiratory consequences of COVID-19 lasted for a median of 4 months in a cohort of children aged 2-18 years of age. Acta Paediatr. 2022 Jun;111(6):1201-6.
19. Gonzalez-Aumatell A, Bovo MV, Carreras-Abad C, Cuso-Perez S, Domenech Marsal E, Coll-Fernandez R, et al. Social, Academic, and Health Status Impact of Long COVID on Children and Young People: An Observational, Descriptive, and Longitudinal Cohort Study. Children (Basel). 2022 Oct 31;9(11).
20. Guido CA, Lucidi F, Midulla F, Zicari AM, Bove E, Avenoso F, et al. Neurological and psychological effects of long COVID in a young population: A cross-sectional study. Front Neurol. 2022;13:925144.
21. Kikkenborg Berg S, Dam Nielsen S, Nygaard U, Bundgaard H, Palm P, Rotvig C, et al. Long COVID symptoms in SARS-CoV-2-positive adolescents and matched controls (LongCOVIDKidsDK): a national, cross-sectional study. Lancet Child Adolesc Health. 2022 Apr;6(4):240-8.
22. Kikkenborg Berg S, Palm P, Nygaard U, Bundgaard H, Petersen MNS, Rosenkilde S, et al. Long COVID symptoms in SARS-CoV-2-positive children aged 0-14 years and matched controls in Denmark (LongCOVIDKidsDK): a national, cross-sectional study. Lancet Child Adolesc Health. 2022 Sep;6(9):614-23.
23. Stephenson T, Pinto Pereira SM, Shafran R, de Stavola BL, Rojas N, McOwat K, et al. Physical and mental health 3 months after SARS-CoV-2 infection (long COVID) among adolescents in England (CLoCk): a national matched cohort study. Lancet Child Adolesc Health. 2022 Apr;6(4):230-9.
24. Adler L, Israel M, Yehoshua I, Azuri J, Hoffman R, Shahar A, et al. Long COVID symptoms in Israeli children with and without a history of SARS-CoV-2 infection: a cross-sectional study. BMJ Open. 2023 Feb 21;13(2):e064155.
25. Ahn B, Choi SH, Yun KW. Non-neuropsychiatric Long COVID Symptoms in Children Visiting a Pediatric Infectious Disease Clinic After an Omicron Surge. Pediatr Infect Dis J. 2023 May 1;42(5):e143-e5.
26. Al-Shamrani A, Al-Shamrani K, Al-Otaibi M, Alenazi A, Aldosaimani H, Aldhalaan Z, et al. Residual Cough and Asthma-like Symptoms Post-COVID-19 in Children. Children (Basel). 2023 Jun 8;10(6).
27. Atchison CJ, Whitaker M, Donnelly CA, Chadeau-Hyam M, Riley S, Darzi A, et al. Characteristics and predictors of persistent symptoms post-COVID-19 in children and young people: a large community cross-sectional study in England. Arch Dis Child. 2023 Jul;108(7):e12.
28. Jamaica Balderas L, Navarro Fernandez A, Dragustinovis Garza SA, Orellana Jerves MI, Solis Figueroa WE, Koretzky SG, et al. Long COVID in children and adolescents: COVID-19 follow-up results in third-level pediatric hospital. Front Pediatr. 2023;11:1016394.
29. Buonsenso D, Morello R, Mariani F, De Rose C, Cortese R, Vetrugno L, et al. Role of Lung Ultrasound in the Follow-Up of Children with Previous SARS-CoV-2 Infection: A Case-Control Assessment of Children with Long COVID or Fully Recovered. Journal of Clinical Medicine. 2023;12(9):3342.
30. Garai R, Krivácsy P, Herczeg V, Kovács F, Tél B, Kelemen J, et al. Clinical assessment of children with long COVID syndrome. Pediatric Research. 2023 2023/05/01;93(6):1616-25.
31. Heiss R, Tan L, Schmidt S, Regensburger AP, Ewert F, Mammadova D, et al. Pulmonary Dysfunction after Pediatric COVID-19. Radiology. 2023 Mar;306(3):e221250.
32. Morello R, Mariani F, Mastrantoni L, De Rose C, Zampino G, Munblit D, et al. Risk factors for post-COVID-19 condition (Long Covid) in children: a prospective cohort study. EClinicalMedicine. 2023 May;59:101961.

33. Camporesi A, Morello R, La Rocca A, Zampino G, Vezzulli F, Munblit D, et al. Characteristics and predictors of Long Covid in children: a 3-year prospective cohort study. EClinicalMedicine. 2024 Oct;76:102815.
34. Paniskaki K, Goretzki S, Anft M, Konik MJ, Meister TL, Pfaender S, et al. Increased SARS-CoV-2 reactive low avidity T cells producing inflammatory cytokines in pediatric post-acute COVID-19 sequelae (PASC). Pediatr Allergy Immunol. 2023 Dec;34(12):e14060.
35. Shmueli E, Bar-On O, Amir B, Mei-Zahav M, Stafler P, Levine H, et al. Pulmonary Evaluation in Children with Post-COVID-19 Condition Respiratory Symptoms: A Prospective Cohort Study. J Clin Med. 2023 Nov 1;12(21).
36. Stephenson T, Pinto Pereira SM, Nugawela MD, McOwat K, Simmons R, Chalder T, et al. Long COVID-six months of prospective follow-up of changes in symptom profiles of non-hospitalised children and young people after SARS-CoV-2 testing: A national matched cohort study (The CLoCk) study. PLoS One. 2023;18(3):e0277704.
37. Pinto Pereira SM, Nugawela MD, Rojas NK, Shafran R, McOwat K, Simmons R, et al. Post-COVID-19 condition at 6 months and COVID-19 vaccination in non-hospitalised children and young people. Arch Dis Child. 2023 Apr;108(4):289-95.
38. Valenzuela G, Alarcón-Andrade G, Schulze-Schiapacasse C, Rodríguez R, García-Salum T, Pardo-Roa C, et al. Short-term complications and post-acute sequelae in hospitalized paediatric patients with COVID-19 and obesity: A multicenter cohort study. Pediatr Obes. 2023;18(2):12.
39. Delogu AB, Aliberti C, Birritella L, De Rosa G, De Rose C, Morello R, et al. Autonomic cardiac function in children and adolescents with long COVID: a case-controlled study. Eur J Pediatr. 2024 May;183(5):2375-82.
40. Foret-Bruno P, Shafran R, Stephenson T, Nugawela MD, Chan D, Ladhani S, et al. Prevalence and co-occurrence of cognitive impairment in children and young people up to 12-months post infection with SARS-CoV-2 (Omicron variant). Brain Behav Immun. 2024 Jul;119:989-94.
41. Stephenson T, Shafran R, Ladhani SN. Long COVID in children and adolescents. Curr Opin Infect Dis. 2022;35(5):461-7.
42. Hersh Z, Weisband YL, Bogan A, Leibovich A, Obolski U, Nevo D, et al. Impact of Long-COVID in children: a large cohort study. Child Adolesc Psychiatry Ment Health. 2024 Apr 15;18(1):48.
43. Hosozawa M, Hori M, Hayama-Terada M, Arisa I, Muto Y, Kitamura A, et al. Prevalence and risk factors of post-coronavirus disease 2019 condition among children and adolescents in Japan: A matched case-control study in the general population. Int J Infect Dis. 2024 Jun;143:107008.
44. Korkmaz MF, Senkan GE, Elmas Bozdemir S, Korkmaz M, Koc I, Oral B. Evaluation of long-term pulmonary functions after COVID-19 infection in children: a longitudinal observational cohort study. J Infect Dev Ctries. 2024 Dec 31;18(12.1):S267-S74.
45. Mizrahi D, Lai JKL, Wareing H, Ren Y, Li T, Swain CTV, et al. Effect of exercise interventions on hospital length of stay and admissions during cancer treatment: a systematic review and meta-analysis. British Journal of Sports Medicine. 2024;58(2):97-109.
46. Motilal S, Rampersad R, Adams M, Goon Lun S, Ramdhanie A, Ruiz T, et al. Randomized Controlled Trials for Post-COVID-19 Conditions: A Systematic Review. Cureus. 2024 Aug;16(8):e67603.
47. Pazukhina E, Rumyantsev M, Baimukhambetova D, Bondarenko E, Markina N, El-Taravi Y, et al. Event rates and incidence of post-COVID-19 condition in hospitalised SARS-CoV-2 positive children and young people and controls across different pandemic waves: exposure-stratified prospective cohort study in Moscow (StopCOVID). BMC Med. 2024;22(1):023-03221.
48. Pinto Pereira SM, Nugawela MD, Stephenson T, Foret-Bruno P, Dalrymple E, Xu L, et al. Post-Covid-19 condition (Long Covid) in children and young people 12 months after infection or

reinfection with the Omicron variant: a prospective observational study. Sci Rep. 2024 Apr 30;14(1):9957.
49. Pinto Pereira SM, Nugawela MD, McOwat K, Dalrymple E, Xu L, Ladhani SN, et al. Symptom Profiles of Children and Young People 12 Months after SARS-CoV-2 Testing: A National Matched Cohort Study (The CLoCk Study). Children (Basel). 2023 Jul 14;10(7).
50. Razzaghi H, Forrest CB, Hirabayashi K, Wu Q, Allen AJ, Rao S, et al. Vaccine Effectiveness Against Long COVID in Children. Pediatrics. 2024 Apr 1;153(4).
51. Mandel H, Yoo YJ, Allen AJ, Abedian S, Verzani Z, Karlson EW, et al. Long COVID Incidence Proportion in Adults and Children Between 2020 and 2024: An Electronic Health Record-Based Study From the RECOVER Initiative. Clin Infect Dis. 2025 Jul 18;80(6):1247-61.
52. Schiavo M, Di Filippo P, Porreca A, Prezioso G, Orlandi G, Rossi N, et al. Potential Predictors of Long COVID in Italian Children: A Cross-Sectional Survey. Children (Basel). 2024 Feb 9;11(2).
53. Setiabudi D, Azhali BA, Tirtosudiro MA, Ramadhan MH, Rinaldhi M, Nataprawira HM. Long COVID or Post-Acute Sequelae of COVID-19 (PASC) in Children and Adolescents. Clin Med Res. 2024 Sep;22(3):131-7.
54. Seylanova N, Chernyavskaya A, Degtyareva N, Mursalova A, Ajam A, Xiao L, et al. Core outcome measurement set for research and clinical practice in post-COVID-19 condition (long COVID) in children and young people: an international Delphi consensus study "PC-COS Children". Eur Respir J. 2024 Mar;63(3).
55. Wang H, Lu F, Ni X, Luo R, Chen L, Yuan J, et al. Acute and persistent symptoms of COVID-19 infection in school-aged children: a retrospective study from China. BMC Public Health. 2024 Feb 1;24(1):344.
56. Chepo M, Martin S, Deom N, Khalid AF, Vindrola-Padros C. Mind the gap: examining policy and social media discourse on Long COVID in children and young people in the UK. BMC Public Health. 2025 Apr 12;25(1):1373.
57. Cianciulli A, Santoro E, Manente R, Pacifico A, Comunale G, Finizio M, et al. Validation of a Questionnaire on the Post-COVID-19 Condition (Long COVID): A Cross-Sectional Study in Italy. Infect Dis Rep. 2025 Jun 11;17(3).
58. Coughtrey A, Pereira SMP, Ladhani S, Shafran R, Stephenson T. Long COVID in children and young people: then and now. Curr Opin Infect Dis. 2025 Oct 1;38(5):487-92.
59. Ford ND, Vahratian A, Pratt CQ, Yousaf AR, Gregory CO, Saydah S. Long COVID Prevalence and Associated Activity Limitation in US Children. JAMA Pediatr. 2025 Apr 1;179(4):471-3.
60. Groohi-Sardou S, Dehghani MM, Raesi R, Hushmandi K, Daneshi S. Determining the Delayed Effects of COVID-19 in Children Hospitalized in Southeastern Iran from 2021 to 2023. Curr Pediatr Rev. 2025 Jul 30.
61. Gross RS, Carmilani M, Stockwell MS. Long COVID in Young Children, School-Aged Children, and Teens. JAMA Pediatr. 2025 May 27.
62. Gupte A, Sriram S, Gunasekaran V, Chaudhari K, Kamat D. The Triad of COVID-19 in Children: Acute COVID-19, Multisystem Inflammatory Syndrome, and Long COVID-Part II. Pediatr Ann. 2025 Jan;54(1):e40-e4.
63. Hussein NR, Khalid RC, Jamal TB, Mahdi SA, Mustafa AS, Mohammed BI, et al. Prevalence of anti-SARS-CoV-2 IgG positivity and long COVID-19 in pediatric age group. J Infect Dev Ctries. 2025 Mar 31;19(3):335-41.
64. Lorman V, Bailey LC, Song X, Rao S, Hornig M, Utidjian L, et al. Pediatric Long COVID Subphenotypes: An EHR-based study from the RECOVER program. PLOS Digit Health. 2025 Apr;4(4):e0000747.

65. Noij LCE, Lap CR, Luijten MAJ, Hashimoto S, Teela L, Oostrom KJ, et al. Quality of life and mental health in children with long COVID. Commun Med (Lond). 2025 Jul 3;5(1):271.
66. Rojas NK, Martin S, Cortina-Borja M, Shafran R, Fox-Smith L, Stephenson T, et al. Health and Experiences During the COVID-19 Pandemic Among Children and Young People: Analysis of Free-Text Responses From the Children and Young People With Long COVID Study. J Med Internet Res. 2025 Jan 28;27:e63634.
67. Terry P, Heidel RE, Wilson AQ, Dhand R. Risk of long covid in patients with pre-existing chronic respiratory diseases: a systematic review and meta-analysis. BMJ Open Respir Res. 2025 Jan 30;12(1).
68. Var SR, Maeser N, Blake J, Zahs E, Deep N, Vasilakos Z, et al. Pulmonary and Immune Dysfunction in Pediatric Long COVID: A Case Study Evaluating the Utility of ChatGPT-4 for Analyzing Scientific Articles. J Clin Med. 2025 Aug 25;14(17).
69. Wee LE, Lim JT, Tan JYJ, Li J, Chiew C, Yung CF, et al. Long-term multi-systemic complications after SARS-CoV-2 Omicron and Delta infection in children: a retrospective cohort study. Clin Microbiol Infect. 2025 Apr;31(4):616-24.
70. Willis MC, Hansen DJ. Commentary: Psychosocial features of pediatric long COVID and the challenges of considering temporal and environmental context during a pandemic. J Pediatr Psychol. 2025 Oct 1;50(10):927-9.
71. Wurm J, Ritz N, Zimmermann P. Coronavirus disease 2019 (COVID-19) in children: Evolving epidemiology, immunology, symptoms, diagnostics, treatment, post-COVID-19 conditions, prevention strategies, and future directions. J Allergy Clin Immunol. 2025;155(4):1071-81.
72. Smane L, Stars I, Pucuka Z, Roge I, Pavare J. Persistent clinical features in paediatric patients after SARS-CoV-2 virological recovery: a retrospective population-based cohort study from a single centre in Latvia. BMJ Paediatr Open. 2020;4(1):e000905.
73. Osmanov IM, Spiridonova E, Bobkova P, Gamirova A, Shikhaleva A, Andreeva M, et al. Risk factors for post-COVID-19 condition in previously hospitalised children using the ISARIC Global follow-up protocol: a prospective cohort study. Eur Respir J. 2022 Feb;59(2).
74. Pazukhina E, Andreeva M, Spiridonova E, Bobkova P, Shikhaleva A, El-Taravi Y, et al. Prevalence and risk factors of post-COVID-19 condition in adults and children at 6 and 12 months after hospital discharge: a prospective, cohort study in Moscow (StopCOVID). BMC Med. 2022 Jul 6;20(1):244.
75. Stephenson T, Pinto Pereira SM, Nugawela MD, Dalrymple E, Harnden A, Whittaker E, et al. A 24-month National Cohort Study examining long-term effects of COVID-19 in children and young people. Commun Med (Lond). 2024 Dec 4;4(1):255.
76. Gross RS, Thaweethai T, Salisbury AL, Kleinman LC, Mohandas S, Rhee KE, et al. Characterizing Long COVID Symptoms During Early Childhood. JAMA Pediatr. 2025 May 27;179(7):781-92.
77. Rao S, Azuero-Dajud R, Lorman V, Landeo-Gutierrez J, Rhee KE, Ryu J, et al. Ethnic and racial differences in children and young people with respiratory and neurological post-acute sequelae of SARS-CoV-2: an electronic health record-based cohort study from the RECOVER Initiative. EClinicalMedicine. 2025 Feb;80:103042.
78. Blomberg B, Mohn KG, Brokstad KA, Zhou F, Linchausen DW, Hansen BA, et al. Long COVID in a prospective cohort of home-isolated patients. Nature Medicine. 2021 Sep;27(9):1607-13.
79. Fink TT, Marques HHS, Gualano B, Lindoso L, Bain V, Astley C, et al. Persistent symptoms and decreased health-related quality of life after symptomatic pediatric COVID-19: A prospective study in a Latin American tertiary hospital. Clinics (Sao Paulo). 2021;76:e3511.

80. Matteudi T, Luciani L, Fabre A, Minodier P, Boucekine M, Bosdure E, et al. Clinical characteristics of paediatric COVID-19 patients followed for up to 13 months. Acta Paediatr. 2021 Dec;110(12):3331-3.
81. Molteni E, Sudre CH, Canas LS, Bhopal SS, Hughes RC, Antonelli M, et al. Illness duration and symptom profile in symptomatic UK school-aged children tested for SARS-CoV-2. The Lancet Child & adolescent health. 2021 Oct;5(10):708-18.
82. Radtke T, Ulyte A, Puhan MA, Kriemler S. Long-term Symptoms After SARS-CoV-2 Infection in Children and Adolescents. JAMA. 2021 Jul 15;326(9):869-71.
83. Say D, Crawford N, McNab S, Wurzel D, Steer A, Tosif S. Post-acute COVID-19 outcomes in children with mild and asymptomatic disease. The Lancet Child & adolescent health. 2021 Jun;5(6):e22-e3.
84. Sterky E, Olsson-Akefeldt S, Hertting O, Herlenius E, Alfven T, Ryd Rinder M, et al. Persistent symptoms in Swedish children after hospitalisation due to COVID-19. Acta Paediatr. 2021 Sep;110(9):2578-80.
85. Bergia M, Sanchez-Marcos E, Gonzalez-Haba B, Hernaiz AI, de Ceano-Vivas M, Garcia Lopez-Hortelano M, et al. Comparative study shows that 1 in 7 Spanish children with COVID-19 symptoms were still experiencing issues after 12 weeks. Acta Paediatr. 2022 Aug;111(8):1573-82.
86. Bloise S, Isoldi S, Marcellino A, De Luca E, Dilillo A, Mallardo S, et al. Clinical picture and long-term symptoms of SARS-CoV-2 infection in an Italian pediatric population. Ital J Pediatr. 2022 May 21;48(1):79.
87. Borch L, Holm M, Knudsen M, Ellermann-Eriksen S, Hagstroem S. Long COVID symptoms and duration in SARS-CoV-2 positive children - a nationwide cohort study. Eur J Pediatr. 2022 Apr;181(4):1597-607.
88. Dumont R, Richard V, Lorthe E, Loizeau A, Pennacchio F, Zaballa ME, et al. A population-based serological study of post-COVID syndrome prevalence and risk factors in children and adolescents. Nat Commun. 2022 Nov 29;13(1):7086.
89. Erol N, Alpinar A, Erol C, Sari E, Alkan K. Intriguing new faces of Covid-19: persisting clinical symptoms and cardiac effects in children. Cardiol Young. 2022 Jul;32(7):1085-91.
90. Funk AL, Kuppermann N, Florin TA, Tancredi DJ, Xie J, Kim K, et al. Post-COVID-19 Conditions Among Children 90 Days After SARS-CoV-2 Infection. JAMA Netw Open. 2022 Jul 1;5(7):e2223253.
91. Guven D, Bulus AD. Clinical and laboratory predictors of long-COVID in children: a single center retrospective study. European review for medical and pharmacological sciences. 2022 Oct;26(20):7695-704.
92. Maddux AB, Berbert L, Young CC, Feldstein LR, Zambrano LD, Kucukak S, et al. Health Impairments in Children and Adolescents After Hospitalization for Acute COVID-19 or MIS-C. Pediatrics. 2022 Sep 1;150(3).
93. Messiah SE, Hao T, DeSantis SM, Swartz MD, Talebi Y, Kohl HW, 3rd, et al. Comparison of Persistent Symptoms Following SARS-CoV-2 Infection by Antibody Status in Nonhospitalized Children and Adolescents. Pediatr Infect Dis J. 2022 Oct 1;41(10):e409-e17.
94. Miller F, Nguyen DV, Navaratnam AM, Shrotri M, Kovar J, Hayward AC, et al. Prevalence and Characteristics of Persistent Symptoms in Children During the COVID-19 Pandemic: Evidence From a Household Cohort Study in England and Wales. Pediatr Infect Dis J. 2022 Dec 1;41(12):979-84.
95. Roessler M, Tesch F, Batram M, Jacob J, Loser F, Weidinger O, et al. Post-COVID-19-associated morbidity in children, adolescents, and adults: A matched cohort study including more than 157,000 individuals with COVID-19 in Germany. PLoS Med. 2022 Nov;19(11):e1004122.

96. Trapani G, Verlato G, Bertino E, Maiocco G, Vesentini R, Spadavecchia A, et al. Long COVID-19 in children: an Italian cohort study. Ital J Pediatr. 2022 Jun 3;48(1):83.
97. Zavala M, Ireland G, Amin-Chowdhury Z, Ramsay ME, Ladhani SN. Acute and Persistent Symptoms in Children With Polymerase Chain Reaction (PCR)-Confirmed Severe Acute Respiratory Syndrome Coronavirus 2 (SARS-CoV-2) Infection Compared With Test-Negative Children in England: Active, Prospective, National Surveillance. Clin Infect Dis. 2022 Aug 24;75(1):e191-e200.
98. Baptista de Lima J, Salazar L, Fernandes A, Teixeira C, Marques L, Afonso C. Long COVID in Children and Adolescents: A Retrospective Study in a Pediatric Cohort. Pediatr Infect Dis J. 2023 Apr 1;42(4):e109-e11.
99. Ertesvag NU, Iversen A, Blomberg B, Ozgumus T, Rijal P, Fjelltveit EB, et al. Post COVID-19 condition after delta infection and omicron reinfection in children and adolescents. EBioMedicine. 2023 Jun;92:104599.
100. Jarupan M, Jantarabenjakul W, Jaruampornpan P, Subchartanan J, Phasomsap C, Sritammasiri T, et al. Long COVID and Hybrid Immunity among Children and Adolescents Post-Delta Variant Infection in Thailand. Vaccines (Basel). 2023 Apr 23;11(5).
101. Korner RW, Bansemir OY, Franke R, Sturm J, Dafsari HS. Atopy and Elevation of IgE, IgG3, and IgG4 May Be Risk Factors for Post COVID-19 Condition in Children and Adolescents. Children (Basel). 2023 Sep 25;10(10).
102. Li J, Nadua K, Chong CY, Yung CF. Long COVID prevalence, risk factors and impact of vaccination in the paediatric population: A survey study in Singapore. Ann Acad Med Singap. 2023 Oct 30;52(10):522-32.
103. Mancino E, Nenna R, Matera L, La Regina DP, Petrarca L, Iovine E, et al. A Single Center Observational Study on Clinical Manifestations and Associated Factors of Pediatric Long COVID. Int J Environ Res Public Health. 2023 Sep 21;20(18).
104. Sedik RNM. The clinical course and outcomes of SARS-CoV-2 virus infection in children: a 24-week follow-up study in Sulaimaniyah, Iraq. BMC Pediatr. 2023 Jun 17;23(1):303.
105. Seery V, Raiden S, Penedo JMG, Borda M, Herrera L, Uranga M, et al. Persistent symptoms after COVID-19 in children and adolescents from Argentina. Int J Infect Dis. 2023 Apr;129:49-56.
106. Warren-Gash C, Lacey A, Cook S, Stocker D, Toon S, Lelii F, et al. Post-COVID-19 condition and persisting symptoms in English schoolchildren: repeated surveys to March 2022. BMC Infect Dis. 2023 Apr 5;23(1):201.
107. Boyarchuk O, Perestiuk V, Kosovska T, Volianska L. Coagulation profile in hospitalized children with COVID-19: pediatric age dependency and its impact on long COVID development. Front Immunol. 2024;15:1363410.
108. Calcaterra V, Tagi VM, D'Auria E, Lai A, Zanelli S, Montanari C, et al. Long-term effects of SARS-CoV-2 infection in hospitalized children: findings from an Italian single-center study. Ital J Pediatr. 2024 Feb 14;50(1):27.
109. Kostev K, Smith L, Koyanagi A, Konrad M, Jacob L. Post-COVID-19 conditions in children and adolescents diagnosed with COVID-19. Pediatr Res. 2024 Jan;95(1):182-7.
110. Sansone F, Di Filippo P, Russo D, Sgrazzutti L, Di Pillo S, Chiarelli F, et al. Lung function assessment in children with Long-Covid syndrome. Pediatr Pulmonol. 2024 Feb;59(2):472-81.
111. Sarani M, Motamed N, Hatami G, Namvar A, Ravanipour M. Long COVID in children and adolescents: a historical cohort study with a population-based control group from Iran. BMC Infect Dis. 2024 Sep 30;24(1):1074.
112. Wongwathanavikrom NB, Tovichien P, Udomittipong K, Palamit A, Tiamduangtawan P, Mahoran K, et al. Incidence and risk factors for long COVID in children with COVID-19 pneumonia. Pediatr Pulmonol. 2024 May;59(5):1330-8.

113. Britton PN, Burrell R, Chapman E, Boyle J, Alexander S, Belessis Y, et al. Post COVID-19 conditions in an Australian pediatric cohort, 3 months following a Delta outbreak. Pediatr Res. 2025 Apr;97(5):1668-75.
114. Dixon BE, Allen KS, Simmons N, Brinkley J, Andrews JG, Dzomba BJ, et al. Incidence of long COVID among U.S. children and adults during the omicron era - Tracking Post-COVID Conditions (Track-PCC) network, 2022-2023. J Infect Public Health. 2025 Nov;18(11):102935.
115. Esposito S, Puntoni M, Deolmi M, Ramundo G, Maglietta G, Poeta M, et al. Long COVID in pediatric age: an observational, prospective, longitudinal, multicenter study in Italy. Front Immunol. 2025;16:1466201.
116. Iijima H, Funaki T, Kubota M. Long-COVID in children and their parents: A prospective cohort study. Pediatr Int. 2025 Jan-Dec;67(1):e70042.
117. Yang J, Tamberou C, Arnee E, Squara PA, Boukhlal A, Nguyen JL, et al. Quantifying all-cause healthcare resource utilization and costs of children with mild-to-moderate long COVID in France. J Med Econ. 2025 Dec;28(1):1002-13.
